# Performance and stability of membrane-photoelectrode assemblies with BiVO_4_ photoanodes for water splitting

**DOI:** 10.1039/d6se00417b

**Published:** 2026-06-19

**Authors:** Roberto Valenza, Sebastiano Gadolini, Isaac Holmes-Gentle, Francesco Spanu, Elena C. Corbos, Sophia Haussener

**Affiliations:** a Laboratory of Renewable Energy Science and Engineering, Institute of Mechanical Engineering, École Polytechnique Fédérale de Lausanne 1015 Lausanne Switzerland sophia.haussener@epfl.ch; b Johnson Matthey Technology Centre Blounts Court Road Reading RG4 9NH UK

## Abstract

Membrane-photoelectrode assemblies are a promising device configuration to directly electrolyse liquid water or water vapour into hydrogen and oxygen using solar energy. For the first time, we studied the performance and the stability of membrane-photoelectrode assemblies with BiVO_4_ photoanodes and CoPi co-catalysts on metallic felt at different temperatures. Upon illumination with simulated solar light, photocurrent densities of 0.23 mA cm^−2^ at 1 V *vs.* RHE were obtained with the proton-exchange ionomer and 0.06 mA cm^−2^ with the anion-exchange ionomer, all using liquid water at 30 °C. Operation with liquid water at 56 °C reduced the onset potential difference under light and in the dark due to more severe recombination of charges, independent of the choice of ionomer. The (photoelectro)chemical corrosion reactions resulted in the dissolution of Bi, V, Mo and Co, which was accelerated by temperature. The dissolved species formed solid particles mainly containing vanadium in the proton-exchange membranes. Operation of proton-exchange membrane-photoelectrode assemblies with water vapour resulted in an 85% decrease in the photocurrent density produced at 1 V *vs.* RHE as the hydration of the ionomer was reduced. The more acidic local pH at the ionomer–photoelectrode interface (compared to liquid water operation) accelerated the dissolution of the photoactive material in time, resulting in a faster decrease in the saturation current density. Membrane-photoelectrode assemblies have been demonstrated to expand practical device configurations beyond conventional planar setups. The remaining performance gap with respect to planar photoelectrodes highlights the gains that systematic optimisation of membrane-photoelectrode assemblies can still unlock.

## Abstract 2

The performance and stability of membrane-photoelectrode assemblies with BiVO_4_ photoanodes and CoPi co-catalysts on metallic felt were assessed by performing water splitting at different temperatures.

## Graphical abstract

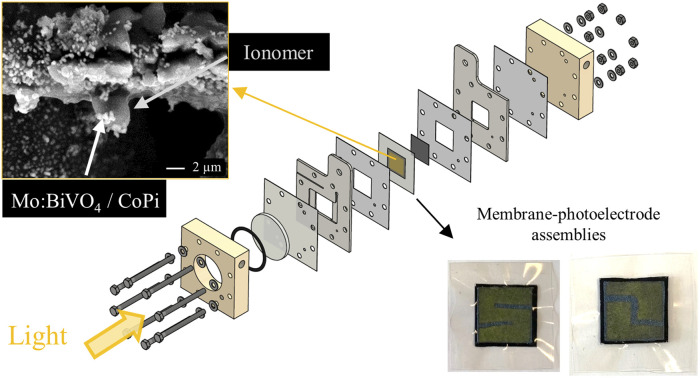

## 1 Introduction

Various device configurations have been proposed for the solar-driven conversion of water into hydrogen and oxygen using a photoelectrochemical (PEC) approach, *i.e.* with at least a semiconductor in direct contact with an electrolyte.^1^ A membrane-photoelectrode assembly (MPEA) is a system in which a solid ionomer is used to ionically connect a semiconductor on a conductive substrate to another electrode.

The use of solid ionomer electrolytes allows the operation of the electrolyzer with pure water reactant feeds, either in the liquid or gas phase. Humidity in air can also be used, making the application attractive for locations with limited access to liquid water. Using humid air, the parasitic optical losses caused by bubble generation in liquid solutions may be avoided at the expense of earlier mass transport limitations.^2^ Whilst hydrated solid ionomers typically have a lower ionic conductivity than concentrated liquid electrolytes,^3^ their low hydrogen and oxygen permeability (in the order of 10^−11^ mol s^−1^ cm^−2^ bar^−1^)^4^ allows for the use of thin membranes (*e.g.*, ∼100 µm thick) and high-purity hydrogen generation. This approach minimizes gaseous product crossover, reduces ohmic losses, and enhances partial load operation efficiency compared to systems using liquid electrolytes and porous separators.^5^ The main disadvantage of solid ionomers is, however, their elevated price which currently limits their attractiveness for large-scale utilization.^6^ Similar to liquid electrolytes, some of the commercial ionomers also parasitically absorb light, potentially reducing the fraction of the solar spectrum reaching the photoelectrodes.

Monopolar solid ionomers operating at low temperature (below 80 °C) are divided into two categories depending on the type of ion which they can selectively transfer: cation-exchange (commonly called proton-exchange) or anion-exchange ionomers.^7,8^ When properly hydrated, proton-exchange ionomers impose an acidic local environment at the interface with the photoelectrode (pH ≈ 2).^9^ The low pH limits the choice of suitable semiconductors and co-catalysts to those that do not suffer from (photo)corrosion, especially for the photoanode performing the oxygen evolution reaction (OER).^10^ Most of the commercial proton-exchange ionomers, *i.e.* Nafion™ of Chemours, are composed of perfluorinated alkylated substances (PFASs), whose production has been recently restricted due to their severe impact on the environment and health.^11^

Anion-exchange ionomers are less developed, as apparent from their shorter lifetimes compared to proton-exchange ionomers.^12^ Good electrical insulating properties are achieved while introducing positively charged functional groups to ensure the transfer of hydroxyl anions when hydrated. Various backbones not necessarily relying on PFASs (*e.g.*, heteroatom-based polyaromatics, poly(arylene)s and polyolefins) can be used.^13^ The local alkaline pH in the nanochannels of anion-exchange ionomers may be beneficial for the stability of a larger number of more common and less expensive materials like hematite (α-Fe_2_O_3_) photoanodes with co-catalysts based on transition metals like Co or Ni.^14,15^

Different metal oxide semiconductors have been tested in the MPEA configuration. Metallic meshes of Ti or W were partially oxidized to create a thin layer of the semiconductor (TiO_2_ or WO_3_, respectively) on their surface and then integrated in proton-exchange MPEAs.^16–18^ Bismuth vanadate (BiVO_4_) was deposited on Ti felt through dip-coating techniques and covered with a proton-exchange ionomer to form MPEAs.^19,20^ A record photocurrent density of 2.1 mA cm^−2^ at 1.23 V *vs.* RHE was obtained with a W-doped BiVO_4_ photoanode on titanium felt under simulated sunlight using a 0.1 M Na_2_SO_4_ aqueous solution. A transparent porous conductive substrate made of interconnected SiO_2_ fibres coated with fluorine-doped tin oxide (FTO) was tested with different semiconducting materials in contact with a proton-exchange ionomer.^21^ Using transparent conductive substrates, a tandem photoanode-photocathode MPEA may be developed by properly tuning the bandgaps of the two materials to utilise a larger fraction of the solar spectrum.^22^ Anion exchange ionomers were used to integrate photoelectrodes made of TiO_2_ or hematite (α-Fe_2_O_3_).^23,24^

The MPEA review by Amano *et al.*^25^ describes the synthesis of photoelectrodes, their integration in an assembly with solid ionomers and their performance characterization using water in the liquid or gas phase. However, there is still limited information on the effects of (photo)corrosion of the semiconductor or of the co-catalysts in this device configuration. Understanding the mechanism and the effects of the degradation phenomena is crucial to cover the stability gap for technologically viable PEC water splitting.^26^

The majority of MPEAs were tested under ambient conditions. Operation of PEC devices at higher temperature may affect light absorption, charge separation and recombination as well as water splitting and (photo)corrosion reaction rates.^27^ The effects of temperature in MPEAs need clarification. Mass transport of water from the channel to the surface of the photoelectrode may be affected by the flow field geometry of the cell, especially if humid air is used as the reactant. Mass transport limitations caused by non-homogeneous relative humidity in the cell have to be avoided while minimizing the pressure drop in the device. This trade-off has been studied in depth for proton-exchange^28,29^ and anion-exchange electrolysis^30^ but it has never been addressed for MPEAs.

In this work, the performance and stability of MPEAs for solar water splitting with a Mo-doped BiVO_4_ (Mo:BiVO_4_) photoanode and a cobalt phosphate (CoPi) co-catalyst on titanium or stainless steel (SS) felt were assessed. BiVO_4_ was selected as a reference material to investigate the effects of the (photo)corrosion in MPEAs as it is a promising semiconductor for PEC water splitting and the mechanism of (photo)corrosion in liquid electrolyte has already been investigated.^31–36^ CoPi is an efficient co-catalyst for BiVO_4_ as it acts as a passivation layer which suppresses surface recombination of photogenerated minority carriers.^37^

The fabrication of the photoelectrodes on metallic felt was first optimized by evaluating the photoelectrochemical performance in a liquid electrolyte. The optimized samples were then integrated in proton-exchange or anion-exchange MPEAs. Their performance and stability were assessed under simulated solar light using liquid water at 30 °C, 43 °C or 56 °C to evaluate for the first time the effects of temperature on MPEAs (Table S1). The effects of (photo)corrosion reactions were evaluated before and after the PEC tests with liquid water by detailed material (photoabsorber, co-catalyst, membrane, *etc.*) and reactant/product analysis (*via* SEM, ICP-MS, XPS, *etc.*). Finally, vapour-phase water splitting in proton-exchange MPEAs was demonstrated at 30 °C. Their performance and stability were assessed with two different flow field configurations to evaluate their impact on the vapour mass transport.

## 2 Experimental

### 2.1 Fabrication of BiVO_4_ photoanodes

The simplified successive ionic layer adsorption and reaction (s-SILAR) procedure to prepare Mo:BiVO_4_ photoanodes on metallic felt was adapted from the literature.^19^ We used either titanium felts (porosity 72%, thickness 250 µm, Fuel Cell Store) or stainless steel (SS) felts (porosity 82%, thickness 500 µm, Bekaert), which were cleaned through three successive ultrasonic rinsing steps in acetone, ethanol and water. The felts were cut into 2 × 2 cm^2^ pieces and then covered with PVC tape leaving an uncovered area of 3.6 cm^2^ on one of the two sides. This PVC-tape mask followed the shape of the flow field geometry used (straight, single serpentine, or interdigitated) to guarantee the direct contact between the flow field/current collector plate and the uncoated felt. The Bi-precursor solution was 40 mL of 0.025 M Bi(NO_3_)_3_·5H_2_O in water and acetic acid in a 19 : 1 volumetric ratio. The V-precursor solution was 40 mL of 0.025 M NH_4_VO_3_ in water. Mo-doped samples were obtained by substituting 1%, 3% or 5% in moles of NH_4_VO_3_ with (NH_4_)_2_MoO_4_ to maintain a 1 : 1 molar ratio of Bi/(V + W) in the two solutions (to theoretically obtain BiV_0.97_Mo_0.03_O_4_). A single s-SILAR cycle consisted of immersing the masked felt in the Bi-precursor solution for 30 s and then in the V-precursor solution for another 30 s with intervals of 60 s between each immersion to dry the felt under flowing N_2_. After the removal of the tape mask, the samples were annealed in a muffle furnace (Carbolite Gero) at 550 °C for 2 h with a heating rate of 2.5 °C min^−1^. Afterwards, the coated felt was immersed in a 1 M KOH aqueous solution for 20 minutes (to remove the unreacted and excess vanadium), rinsed with water and dried in air. The Mo:BiVO_4_ samples were finally annealed in a retort furnace (Carbolite Gero) at 300 °C for 10 minutes using a 5 °C min^−1^ ramp rate with a flow of 500 mL min^−1^ of a 5% H_2_/95% N_2_ mixture. This mild heat treatment in a reductive hydrogen atmosphere was used to improve the carrier mobility and lifetime in Mo:BiVO_4_.^38^

### 2.2 CoPi co-catalyst photoelectrodeposition

The procedure for CoPi photoelectrodeposition on Mo:BiVO_4_ photoanodes was adapted from the literature^39^ to preferentially deposit the co-catalyst on the most photoactive sites of the semiconductor surface (*i.e.* the exposed surface of the semiconductor). Mo:BiVO_4_ on metallic felt (working electrode), a reversible hydrogen electrode (Gaskatel Hydroflex), and a graphite rod (counter electrode) were immersed in an electrolyte of 0.5 mM Co(NO_3_)_2_·6H_2_O and 0.1 M potassium phosphate solution with pH ≈ 7 (molar concentration ratio KH_2_PO_4_ : K_2_HPO_4_ of 1.63 : 1). Using a methodology previously described,^40^ the samples were placed at a distance of 106 mm from a blue LED (ILS OSLON SSL4, peak wavelength 442 nm, 173 ± 7 W m^−2^ at 106 mm) to simulate the useful irradiation absorbed from the AM 1.5G spectrum. The LED was powered by constant-current LED drivers (0.7 A, ILS IZC070) and attached to an aluminium heat sink by using a thermal adhesive. A simplified schematic of the setup is shown in Fig. S1. A constant potential of 1.0 V *vs.* RHE was applied for 20 minutes using a Biologic VSP potentiostat.

### 2.3 Preliminary PEC tests for photoanode optimization

The Mo dopant molar concentration, total semiconductor loading, and CoPi catalyst deposition were optimized by varying the number of s-SILAR cycles and concentration of precursors in order to maximize the photoelectrochemical performance. The PEC measurements were performed in a beaker with 200 mL of a 0.2 M Na_2_SO_4_ aqueous solution and the same setup shown in Fig. S1 was used. Cyclic voltammetry (CVs) between 0.2 and 1.6 V *vs.* RHE at a scan rate of 10 mV s^−1^ was performed, reporting the average of the third forward sweeps of the CV of two different coated felt. The high-frequency resistance (HFR) was obtained by fitting the electrochemical impedance spectroscopy imposing a sinusoidal wave with amplitude of 20 mV and a frequency of 50 kHz at a constant potential of 1.23 V *vs.* RHE. Where specified, 0.1 M of Na_2_SO_3_ was added to the electrolyte as a hole scavenger. The catalytic efficiency *η*_cat_ was used as a performance indicator as previously proposed by Dotan *et al.*^41^ (eqn (1)).1
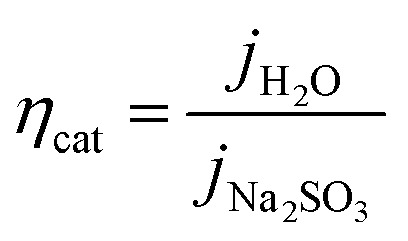


*j*_H_2_O_ is the current density resulting from an experiment without a hole-scavenger (*i.e.* the oxygen evolution reaction will dominate) and *j*_Na_2_SO_3__ is the current density measured with a hole scavenger, Na_2_SO_3_, assuming that all photogenerated holes reaching the surface of the photoelectrode contribute to sulfite anion oxidation without recombination.

### 2.4 Membrane-photoelectrode assembly preparation

The proton-exchange ionomer dispersion was obtained using Nafion™ ionomer pellets, ethanol and deionized water in a 18.5 : 80 : 1.5 mass proportion. For the anion-exchange ionomer, Fumion™ FAA-3 shredded films and 1-propanol in a 25 : 75 mass proportion were used. Layers of proton-exchange and anion-exchange ionomer inks, with thicknesses of 300 µm and 650 µm respectively, were obtained through a doctor blade method. The coated Ti felt was placed in the proton-exchange ionomer layer leaving the semiconductor side on top. For the coated SS felt, the semiconductor side was pointing downwards when placing them in the anion-exchange ionomer layer. The samples were dried in ambient air for 30 minutes to let the solvents evaporate. The excess ionomer was cut away from the dry layers to obtain the ionomer-coated felt. Nafion™ 115 proton-exchange membranes (PEM, thickness 127 µm) coated with a Pt/C cathode catalyst layer (catalyst loading 0.08 mg cm^−2^, Johnson Matthey) and Fumasep™ FAA-3-50 anion-exchange membranes (AEM, thickness 50 µm) coated with a Pt/C layer (catalyst loading 0.4 mg cm^−2^, Johnson Matthey) were used for the membrane-cathode assemblies. To obtain a membrane-photoelectrode assembly, the ionomer-coated felt was placed on top of the catalyst-coated membrane with the semiconductor side pointing upwards and in between some PTFE gaskets. Hot pressing for 2 minutes at 150 °C for a proton-exchange membrane (PEM) or at 80 °C for an anion-exchange membrane (AEM) was performed without applying any pressure, *i.e.* the plates were only touching the two external gaskets without any air gap in between, not to risk compressing too much and damaging the coated porous layer. A simplified schematic of the steps to prepare the membrane-photoelectrode assemblies is shown in Fig. S2.

### 2.5 Photo-electrolyzer cell design

**Fig. 1:**
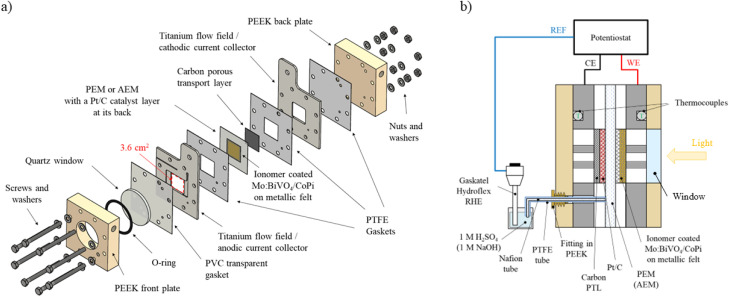
(a) Exploded view of the photo-electrolyzer cell; (b) schematic representation of the system to introduce the external hydrogen reference electrode in an edge-type configuration.

Inspired by low-temperature electrolyzers, a cell to test membrane-photoelectrode assemblies was designed and is henceforth referred to as the photo-electrolyzer (Fig. 1(a)). A quartz window and a PVC gasket transparent in the visible region allowed the illumination of the photoanode integrated in the MPEA. A carbon porous transport layer (Sigracet 39AA, 280 µm thickness, 80% porosity, Fuel Cell Store) was used at the cathode side. Titanium flow fields with straight, single serpentine or interdigitated geometry were also the current collectors in contact with the porous conductive layers. PEEK external plates and PTFE gaskets completed the cell, avoiding leakages.

### 2.6 Introduction of the external reference electrode

An external reference electrode was introduced in the photo-electrolyzer in an edge-type configuration taking inspiration from previously proposed methodologies.^42–44^ A hydrogen reference electrode (Gaskatel Hydroflex) was placed outside the cell and immersed in a 1 M H_2_SO_4_ aqueous solution for PEM experiments. The solution was ionically connected to the membrane through a Nafion™ tube (0.84 mm OD, Fuel Cell Store) in a PTFE tubing (0.9 mm OD) filled with a 1 M H_2_SO_4_ aqueous solution. A 1 M NaOH aqueous solution was alternatively used for the AEM experiments. The PTFE tube was inserted in a Fidex F-130X fitting and inserted in the photo-electrolyzer cathodic PEEK plate to avoid leakages. The schematic representation of the system is shown in Fig. 1(b). The applied potentials *vs.* RHE were not corrected for the ohmic drop in the membrane given the low operating current densities. The Donnan potential at the membrane-liquid electrolyte interface was assumed to be negligible.

### 2.7 Setup for the tests with liquid water or humid air

Liquid deionized water (200 mL) was stored in glass bottles and it was sent to both sides of the photo-electrolyzer using a multi-channel Shenchen LabV1 peristaltic pump with a MC12 pump head. A water volumetric flow rate of 13 mL min^−1^ was imposed. The glass bottles were placed in a 28 L water bath (Fisherbrand Isotemp) to control the temperature of the working fluid. Tests with water in the vapour phase were performed by imposing a flow of nitrogen to pass through a humidifier before reaching the photo-electrolyzer. Nitrogen volumetric flow rates from 20 to 220 mL min^−1^ in both compartments of the cell were imposed through mass flow controllers (Bronkhorst F-201CV). The temperature in the cell was measured using K-type thermocouples connected to a Pico Technology TC-08 data logger. The thermocouples were introduced into a port of each flow field plate to obtain an estimate of the operating temperature of the electrodes. The photo-electrolyzer was illuminated by using AM 1.5G simulated irradiation from a Trisol Solar Simulator (AOI) calibrated with an Ocean Insight FLAME-S-XR1 spectrometer (optical resolution 1.7 nm FWHM) connected to an optical fibre (core diameter 300 µm, length 1 m) and a cosine corrector (CC-3-UV-S, OceanInsight). The schematic representation of the two setup configurations is reported in Fig. S3 and S4.

### 2.8 PEC characterization of the membrane-photoelectrode assemblies

The membrane-photoelectrode assemblies were characterized in the three-electrode configuration by connecting the anodic metallic plate of the photo-electrolyzer to the working electrode, the cathodic plate to the counter electrode and using the external hydrogen electrode as the reference (Fig. 1(b)). The external reference electrode used was considered as the reversible hydrogen electrode (RHE) as in previous reports.^42,45^ The current density–potential characteristic curves of proton-exchange MPEAs were obtained by applying potentials from 0.7 to 1.6 V *vs.* RHE for 60 s. Similar curves were obtained for the anion-exchange MPEAs applying potentials from 0.7 to 1.3 V *vs.* RHE for 60 s as a dark current associated with the oxidation of the SS felt was observed for larger potentials.^46^ The current densities reported in the characteristic curves are the averages of the steady state values of two different samples. All the error bars reported were obtained from the standard deviation of the measurements of two samples. Chronoamperometric measurements imposing 1.23 V *vs.* RHE were interspersed with current density–potential characteristic curves. Tests with liquid water at 30 °C, 43 °C and 56 °C were performed, and only tests at 30 °C were performed with water vapour. The onset potential was defined as the potential *vs.* RHE measured at 0.02 mA cm^−2^ and it was calculated with a linear interpolation of the experimental points. The saturation current density was defined as the average current density measured at 1.2 V *vs.* RHE in the characteristic curves.

Photostability was analysed by extracting 10 mL of liquid water from the anodic reservoir after two current density-potential characterization studies performed after different time spans of chronoamperometry. The chemical stability of the samples was analysed leaving both the proton-exchange or anion-exchange MPEAs, both the metallic felt (Ti and SS) coated with Mo:BiVO_4_ and CoPi (without ionomer), or the annealed SS felt (without any coating), either in liquid water, in a 0.005 M H_2_SO_4_ aqueous solution (pH ≈ 2) or in a 0.01 M NaOH aqueous solution (pH ≈ 12). Liquid samples were then analysed using inductively coupled plasma mass spectroscopy (ICP-MS, Agilent Technologies LC Infinity II Triple Quad 8900) to quantify the amount of dissolved elements.

### 2.9 Material characterization

The Mo:BiVO_4_ coated felt and the MPEAs were observed before and after operation using scanning electron microscopy (SEM). A Jeol JSM-IT300 SEM operating at 20 kV was used for pristine samples, and a Zeiss GeminiSEM 300 working at 3 kV for aged samples. For the cross-section SEM images of pristine MPEAs, the samples were embedded in epoxy resin and mechanically polished after solidification until a mirror-like surface was obtained. For the aged samples, the membranes were broken into two pieces in liquid nitrogen to obtain a sharp rupture. A carbon deposition pre-treatment was performed before every cross-section SEM observation. The nanostructures of Mo:BiVO_4_ were mechanically scratched away from the metallic felt with a razor blade and deposited on a standard Cu grid. They were then observed by transmission electron microscopy (TEM, Tecnai Osiris operated at 200 kV) and energy-dispersive X-ray spectroscopy (EDXS). Anion-exchange membranes after the PEC tests with liquid water at 30 °C were embedded in epoxy and cut by ultra-microtomy into 90 nm lamellae and placed on CF200-CU-50 grids for TEM and EDXS measurements.

The elemental surface composition of the samples was evaluated by X-ray photoelectron spectroscopy (XPS). The synthesis steps from BiVO_4_ to Mo:BiVO_4_ with CoPi on Ti felt were analysed using a Thermo Nexsa instrument, while the effects of photocorrosion were investigated using a Kratos AXIS Supra. The binding energies were corrected with reference to the adventitious C 1s peak at 284.8 eV and normalized separately for the samples measured with each instrument.

Spectral hemispherical reflectance and transmittance spectra of Mo:BiVO_4_ on titanium and SS felt or on a planar FTO-coated glass substrate were measured with a Shimadzu UV-2600 UV-vis-NIR spectrophotometer with an integrating sphere (ISR-2600 PLUS Shimadzu). Absorption spectra were calculated from these data. The Tauc plot was fitted assuming an indirect optical transition to obtain the optical bandgap of the semiconductor at ambient temperature.^47^ A correction for a baseline of the absorbing metallic felt was applied as previously proposed.^48^ The transmittance of a thin layer of a proton-exchange thin layer (10 µm) and anion-exchange (60 µm) ionomer on FTO-coated glass was obtained following the same methodology.

## 3 Results and discussion

### 3.1 Optimization and characterization of the photoanodes

In order to maximize the MPEA performance, the concentration of the Mo dopant and the number of s-SILAR cycles were optimized. Mo acts as an electron donor in the BiVO_4_ lattice.^49^ The optimal molar concentration of the Mo dopant was found to be 3% (Fig. S5). Lower Mo concentrations seem to not provide enough free electrons in the lattice whereas too large Mo concentrations reduce the space charge region thickness and may induce lattice strain or even the formation of Mo oxides and defects, acting as recombination centres.^50^ The optimal number of s-SILAR cycles for Mo:BiVO_4_ on titanium and stainless steel felt was found to be 8 and 6, respectively (Fig. S6, S7(c) and (d)). A current density of 0.57 and 0.69 mA cm^−2^ was achieved at 1.23 V *vs.* RHE with the Ti and SS substrates, respectively. SS felt also allowed for a better extraction of photogenerated charges at lower potentials. For a lower number of s-SILAR cycles, the small amount of semiconductor on the felt leads to reduced light absorption^51^ (Fig. S7(a) and (b)). The high-frequency resistance (HFR) of the samples did not vary on increasing the number of cycles above the optimum (12 Ω cm^2^ for Mo:BiVO_4_ on Ti felt and 20 Ω cm^2^ for the semiconductor on SS felt for more cycles than the optimal one), excluding ohmic losses as an explanation. It is likely that, a larger amount of the generated charge carriers is further away from the interface and charge extraction becomes more difficult. This optimal Mo concentration and number of s-SILAR cycles were fixed for all of the subsequent experiments.

**Fig. 2:**
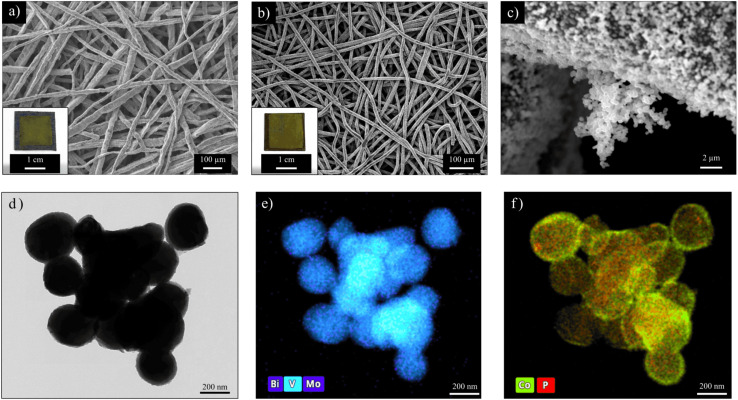
(a) SEM image and photograph of Mo:BiVO_4_ on Ti felt after 8 s-SILAR cycles; (b) SEM image and photograph of Mo:BiVO_4_ on stainless steel felt after 6 s-SILAR cycles; (c) SEM image of a nanostructure of Mo:BiVO_4_ formed on a Ti fibre; (d) TEM image of a Mo:BiVO_4_ with a CoPi nanostructure scratched off a metallic felt; (e) and (f) EDXS elemental mapping of (e) Bi, V and Mo and (f) Co and P of the same nanostructure.

For the optimal number of s-SILAR cycles, the Mo:BiVO_4_ semiconductor nearly completely covers the fibres of both the Ti and SS felt (Fig. 2(a) and (b)). The semiconductor formed nanostructures on the fibres (Fig. 2(c) and S8), increasing the specific surface area for the anodic reaction. Nanostructures also ensure the collection of photogenerated minority charge carriers perpendicular to the direction of light absorption, which is beneficial considering the short diffusion length of doped BiVO_4_ photoanodes (between 50 and 100 nm).^52,53^ After the photoelectrodeposition of CoPi, the co-catalyst formed a layer of approximately 30 nm on Mo:BiVO_4_, helping to suppress the surface recombination at the interface with the electrolyte^37^ without considerably compromising the light absorption (Fig. 2(d)–(f) and S9). The indirect optical bandgap of the semiconductor was measured to be 2.47 eV (Fig. S10), in agreement with values around 2.4–2.5 eV previously reported.^47^

**Fig. 3:**
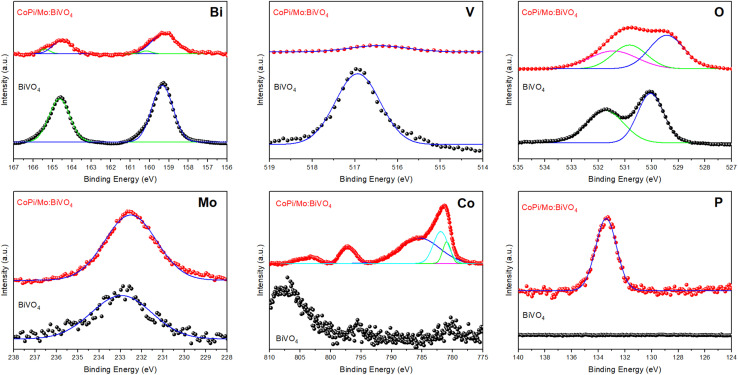
XPS spectra of pristine BiVO_4_ (black dots) and Mo:BiVO_4_ with CoPi (red dots) on titanium felt with the corresponding Lorentzian deconvolution for data fitting (lines) in the binding energy ranges for Bi, V, O, Mo, Co and P.

The surface composition and electronic structure of the photoanodes were analysed by XPS. Pristine BiVO_4_ and Mo:BiVO_4_ with CoPi, both on Ti felt, were initially studied (Fig. 3). For pristine BiVO_4_ on Ti, the Bi spectrum exhibited two peaks at binding energies of 159.2 eV and 164.5 eV, which correspond to Bi 4f_7/2_ and Bi 4f_5/2_ of Bi 4f, attributed to the presence of the Bi(III) state at the surface of the film.^54^ The peak at 516.9 eV was ascribed to the V 2p_3/2_ state of the V(v) oxidation state.^19^ The deconvolution of the O 1s spectrum shows two peaks at 530.0 eV and at 531.7 eV, which were attributed to the oxygen in the lattice and the chemisorbed or dissociated oxygen from water molecules.^54^ The comparison between the spectra of pristine BiVO_4_ and those of Mo:BiVO_4_ with CoPi reveals shifts in the binding energy of the peaks and the variation of their intensities, indicative of strong interactions between the co-catalyst, the semiconductor and the metallic substrate.^55^ The XPS peaks of Bi 4f and V 2p in Mo:BiVO_4_ with CoPi slightly shifted to lower binding energies and decreased in intensity compared to those of the pristine material without a co-catalyst. These shifts suggest charge redistribution and possible catalyst-semiconductor interfacial effects, which can play a critical role in the catalytic performance of the system. The reduction of the intensity of the peaks of Bi and V is in agreement with the TEM results where a conformal layer of CoPi was observed over BiVO_4_ (Fig. 2(e) and (f)). Indeed, similar results have been previously reported in studies examining the surface coverage of co-catalysts on different supports.^56,57^ The O 1s spectrum exhibits noticeable broadening and different peak positions upon co-catalyst deposition. The peaks observed at 529.4 eV, 531.4 eV and 530.8 eV can be deconvoluted into three curves related to lattice oxygen, oxygen vacancies and absorbed OH groups.^58–60^ The appearance of the Mo 3d_5/2_ peak at 232.5 eV confirmed the presence of the Mo dopant in the BiVO_4_ lattice.^61^ However, a minor contribution of Mo was also found in the pristine material, and this was attributed to its presence in traces in the commercial metallic substrate used. After the photo-electrodeposition of CoPi, Co 2p_3/2_ and Co 2p_1/2_ peaks were detected at 780.9 eV and 796.7 eV, respectively. These peaks were attributed to the Co(ii)/Co(iii) oxidation states of cobalt, as previously observed for CoPi. Additional peaks of Co 2p were attributed to the formation of Co–O and Co–O–P interactions.^55,62^ The peak at 133.4 eV in the P 2p region is derived from phosphate, confirming the formation of CoPi on the photo-electrode surface.^54^ Fig. S11 presents the XPS spectra of the as-prepared BiVO_4_ and Mo:BiVO_4_ with CoPi samples on stainless steel felt. With a different substrate, the chemical elements and their oxidation states were the same as those observed using Ti felt. The main differences were attributed to using stainless steel as a metallic support. The presence of Bi, O, Mo, Co, and P species in the metal alloy was confirmed based on the XPS peaks of the annealed stainless steel felt (Fig. S12).

Testing the samples on Ti felt in a 0.2 M Na_2_SO_4_ aqueous solution, the reduction of ohmic losses with the introduction of the Mo dopant and the improvement in the extraction of photogenerated holes with the CoPi co-catalyst can be observed (Fig. S13). A current density of 1.5 mA cm^−2^ was obtained at 1.23 V *vs.* RHE at 30 °C in the optimal configuration. This current is smaller than the maximum photocurrent density obtained with nanostructured planar BiVO_4_ substrates of 7.15 and 7.0 mA cm^−2^ at 1.23 V *vs.* RHE under AM 1.5 G illumination, respectively obtained by Zhao *et al.*^63^ and Liu *et al.*^64^ Despite being still far from the maximum theoretical limit of 7.5 mA cm^−2^, the performance in Na_2_SO_4_ electrolyte reported here is similar to the one of other BiVO_4_ photoanodes on porous substrates previously reviewed by Zafeiropoulos *et al.*^19^ After the photoelectrochemical deposition of CoPi on Mo:BiVO_4_ on Ti felt, the catalytic efficiency *η*_cat_ of the samples increased from 17% to 62% at 0.8 V *vs.* RHE. In the dark, the oxidation of Na_2_SO_3_ led to a dark current density with an onset potential at approximately 0.9 V *vs.* RHE which did not allow the calculation of the catalytic efficiency *η*_cat_ for larger potentials.

### 3.2 Membrane-photoelectrode assemblies

**Fig. 4:**
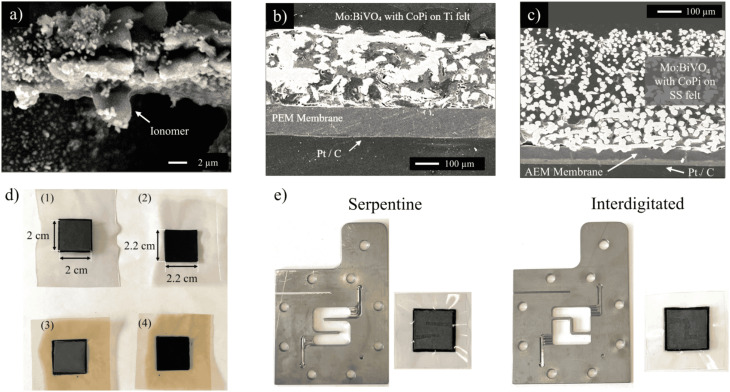
(a) SEM image of the Mo:BiVO_4_ nanostructures on a Ti fibre covered by a proton-exchange ionomer; (b) cross-section SEM images of the proton-exchange membrane-photoelectrode assembly after the hot press treatment; (c) cross-section SEM image of an anion-exchange MPEA after the hot press treatment; (d) photograph of the proton-exchange (or anion-exchange at the bottom) membrane-photoelectrode assemblies: (1)/(3) from the photoanode side or (2)/(4) from the Pt/C cathode catalyst layer side; (e) photograph of the serpentine and interdigitated flow field plates with proton-exchange MPEAs with selective deposition of Mo:BiVO_4_ to follow their shape.

After the deposition of the ionomers and the evaporation of the solvent, the polymer conformally coated the metallic fibres and the nanostructures of Mo:BiVO_4_ with CoPi (Fig. 4(a) and S14). The doctor blade technique facilitated a strategically non-uniform deposition of ionomers throughout the thickness of the felt (Fig. S15). The ionomer only covered the fibres of the felt and the semiconductor nanostructures on the side directly exposed to light, which ensured the electrolytic contact with the photoactive particles and optimal mass transfer of the reactant to the surface of the photoelectrode. The proton-exchange ionomer is almost transparent at wavelengths smaller than the one characteristic of the bandgap of BiVO_4_ (Fig. S16). The anion-exchange ionomer reduces its transmittance in the UV region, which may penalize the generation of charges in the semiconductor. On the opposite side in contact with the membrane, the ionomer filled the pores among the fibres to guarantee a better adhesion to the membrane. The hot press treatment resulted in the integration of the felt in the membrane-photoelectrode assemblies with the Pt/C cathode catalyst layers (Fig. 4(b)–(d)). The use of PVC tape to mask areas where s-SILAR deposition is undesirable enabled the creation of MPEAs which follow the shape of the flow field plate used. This avoids contact between the semiconductor-coated felt and the metal plate contact, which would unfavourably increase contact resistance (although a thin layer of metal oxide may have formed in those regions during the annealing process). Furthermore, preventing the deposition on the non-illuminated electrode area minimizes the wasted photo-active and catalyst materials and optimizes their loading on the felt (Fig. 4(e)).

### 3.3 PEC characterization with liquid water

**Fig. 5:**
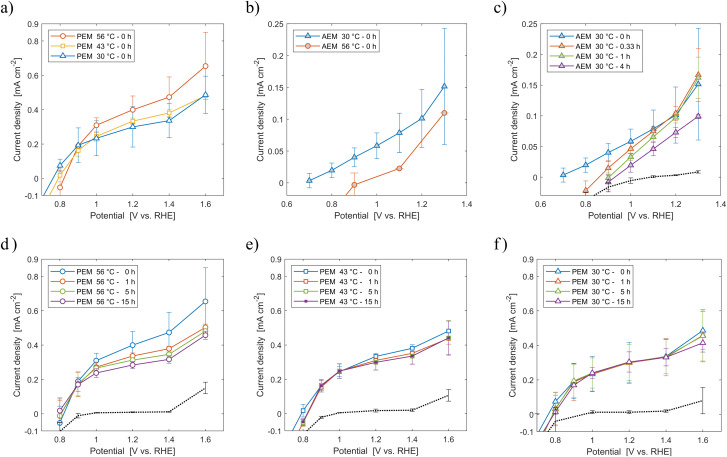
(a) and (b) Current density–potential characteristic curves with liquid water at different temperatures of pristine (a) proton-exchange MPEAs, and (b) anion-exchange MPEAs. (c) Current density–potential characteristic curves of anion-exchange MPEAs after different durations of exposure by chronoamperometry at 1.23 V *vs.* RHE with liquid water at 30 °C. (d)–(f) Current density–potential characteristic curves of proton-exchange MPEAs after different durations of exposure by chronoamperometry at 1.23 V *vs.* RHE with liquid water at (d) 56 °C, (e) 43 °C, and (f) 30 °C. The black dotted lines in the figure represent the characteristic curves with liquid water of the pristine MPEAs in the dark.

The proton-exchange and anion-exchange MPEAs were initially tested at various temperatures using liquid-phase water. For pristine proton-exchange MPEAs, an increase in the onset potential with a temperature increase was observed: 0.77 V *vs.* RHE at 30 °C to 0.83 V *vs.* RHE at 56 °C, with an average rate of +3.7 mV K^−1^ (Fig. 5(a)). The onset potential difference under light and in the dark decreased with increasing temperature, primarily due to a more pronounced surface recombination of photogenerated minority carriers compared to their transfer to the electrolyte, as previously observed for tin-doped hematite photoanodes.^40^ The saturation current density increased from 0.30 mA cm^−2^ at 30 °C to 0.40 mA cm^−2^ at 56 °C (average rate +0.62 µA cm^−2^ K^−1^), following a trend in agreement with previous reports about BiVO_4_ thin films.^61,65^ We expect the bandgap of the semiconductor to decrease with temperature as described by the Varshni model^66^ by approximately 8 meV.^67^ This decrease corresponds to a 1.2% larger photon flux with energy greater than the bandgap. The spectral absorption coefficient at photon wavelengths smaller than the absorption edge increases with temperature. The increased absorption of photons combined with the better kinetics of electrochemical oxidation are expected to lead to a higher saturation current density with increasing temperature.

The pristine anion-exchange MPEAs at 30 °C had an onset potential of 0.81 V *vs.* RHE (Fig. 5(b)), which is comparable to the ones obtained with proton-exchange MPEAs. However, lower photocurrent densities were observed, especially at low applied potentials. At 1 V *vs.* RHE and 30 °C, anion-exchange MPEAs generated a current density of only 0.06 mA cm^−2^, compared to 0.23 mA cm^−2^ with proton-exchange MPEAs under identical conditions. A less efficient charge extraction at the photoanode–electrolyte junction may explain this loss of current at larger applied potentials, further widening the performance gap with respect to the best performing nanostructured photoelectrodes on planar FTO-coated glass.^63,64^ The Fumion™ FAA-3 ionomer partially absorbs light in the visible spectrum and mainly in the UV region, as reported in Fig. S15. Its thickness in the order of hundreds of nm on the photoanode surface is expected not to significantly penalize light absorption in the semiconductor. The onset potential difference under light and in the dark and photocurrent density produced by the anion-exchange MPEAs decreased further when operating at 56 °C.

Next, the behaviour of both proton-exchange and anion-exchange MPEAs was assessed after operating for a certain number of hours. After applying a potential of 1.23 V *vs.* RHE for 4 hours at 30 °C (Fig. S17(a)), both the saturation photocurrent density and onset potential difference produced in anion-exchange MPEAs decreased over time (Fig. 5(c)). On applying the same potential to the proton-exchange MPEAs, a decrease in the saturation current density in time was also observed for samples tested at 56 °C and 43 °C (Fig. 5(d) and (e)). A larger drop in current density was observed in the first hour of the test with a slower subsequent decrease (Fig. S17(b)). The chemical and photoelectrochemical corrosion of BiVO_4_ in an acidic environment followed by the dissolution of the produced species may reduce the amount of the photoactive semiconductor over time,^35^ decreasing the amount of light collected and therefore the saturation photocurrent density. During the tests at 30 °C, the saturation current density was not affected by the application of 1.23 V *vs.* RHE for 15 hours (Fig. 5(f)). The same trends at different temperatures were also observed during chronoamperometry (Fig. S17(b)): the average reduction of the current density in time at 56 °C was −8.6 µA cm^−2^ h^−1^ while it was negligible at 30 °C. The almost constant photocurrent density at 30 °C for 15 h does not imply a perfect stability of the samples; long-term tests (up to thousands of hours) have to be performed to fully evaluate the impact of photocorrosion. The negative currents measured are associated with reduction reactions as previously observed by Zafeiropoulos *et al.*^19^ Their exact nature was not determined but could be associated with the residual oxygen reduction on the uncoated Ti surface, and the reduction of TiO_2_, other oxides or cobalt in CoPi.

### 3.4 Photocorrosion in proton-exchange MPEAs after tests with liquid water

**Fig. 6:**
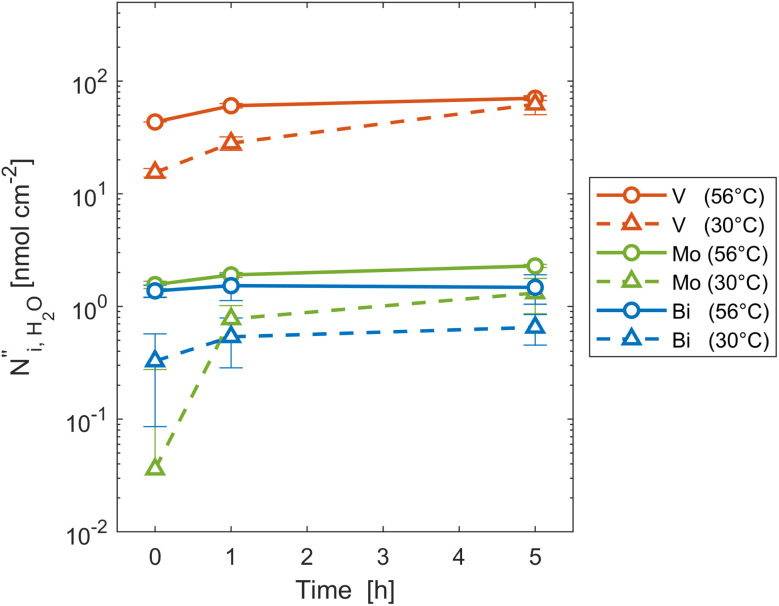
Moles of Bi, V or Mo dissolved in liquid water per illuminated geometric area of the felt after the chronoamperometry performed at different temperatures with proton-exchange MPEAs. The liquid samples were extracted after two potential–current density characteristic curves with simulated solar light.

ICP-MS measurements were used to quantify the amount of various elements in liquid water and thereby evaluate the photocorrosion of Mo:BiVO_4_ photoanodes with CoPi co-catalysts during operation. Following the tests with proton-exchange MPEAs, the liquid water of the anodic reservoir contained more vanadium than bismuth (Fig. 6), with a difference in moles of almost two orders of magnitude (
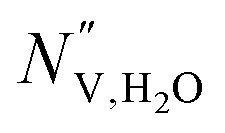
 (5 h, 30 °C) = 62.2 nmol cm^−2^*versus*
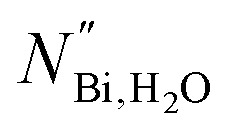
 (5 h, 30 °C) = 0.65 nmol cm^−2^). From the Pourbaix diagram of BiVO_4_, BiO^+^ and VO^−^_4_ are predicted to be produced from the photocorrosion of the material in a mildly acidic environment and under high potential.^31^ The lower concentration of bismuth may be caused by the transfer of the produced BiO^+^ to the cathodic water reservoir through the proton-exchange membrane and the repulsion towards the anodic reservoir of VO^−^_4_ from the negative sulfonic groups of the Nafion™ ionomer. A more complex mechanism governing selective vanadium dissolution, as observed by Lee and Choi,^32^ could also cause such a difference in concentration. In this case, the dissolution of vanadium from BiVO_4_ could be reduced through the use of a V^5+^-saturated electrolyte. At the end of the tests, the amount of Bi measured in liquid water was even less than Mo (
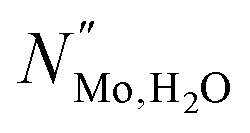
 (5 h, 30 °C) = 1.32 nmol cm^−2^), although the theoretical ratio in the material should be 100 : 3.

It has previously been shown that cobalt phosphate is chemically unstable in an acidic environment.^68^ Surprisingly, no cobalt was detected in the anodic reservoir after the PEC tests with proton-exchange MPEAs, *i.e.* the concentration of Co in water was below the detection limit of 0.05 µg L^−1^. We hypothesize that the catalyst may therefore have dissolved extremely quickly and may have passed to the cathodic reservoir. The performance in 0.2 M Na_2_SO_4_ aqueous solution of the pristine proton-exchange MPEA with blue LED light is indeed similar to that of Mo:BiVO_4_ on Ti felt without CoPi in liquid water (Fig. S18), confirming that the complete dissolution of the thin layer of CoPi may have occurred before the end of this test. Operation at 56 °C accelerates the dissolution of species and the transfer to liquid water without modifying the relative molar ratios between different species observed at 30 °C. Similar trends are observed when comparing photoelectrochemical and chemical dissolution of the species from proton-exchange MPEAs (Fig. S19 (a)–(c)). The applied electrical bias and light increase the quasi-Fermi level of holes in the photoanode *vs.* RHE, accelerating the dissolution of species and their transfer to water without changing the relative molar ratios among the species. However, the dissolution of V and Mo appeared to mainly be chemical rather than photoelectrochemical. On placing a Ti felt coated with Mo:BiVO_4_ and CoPi (without ionomers) in a 0.005 M H_2_SO_4_ aqueous solution with pH ≈ 2, the chemical dissolution at 30 °C of Bi, V and Mo followed the theoretical stoichiometric ratios in the material (Fig. S20(a)). This result confirms that the proton-exchange ionomer may influence not only the transfer of ions but also the photocorrosion mechanism at the photoanode–electrolyte interface. Cobalt was only detected in the chemical stability test with Ti felt coated with Mo:BiVO_4_ and CoPi (without ionomers) in a 0.005 M H_2_SO_4_ aqueous solution, confirming the chemical instability of the co-catalyst (Fig. S21(a)).

**Fig. 7:**
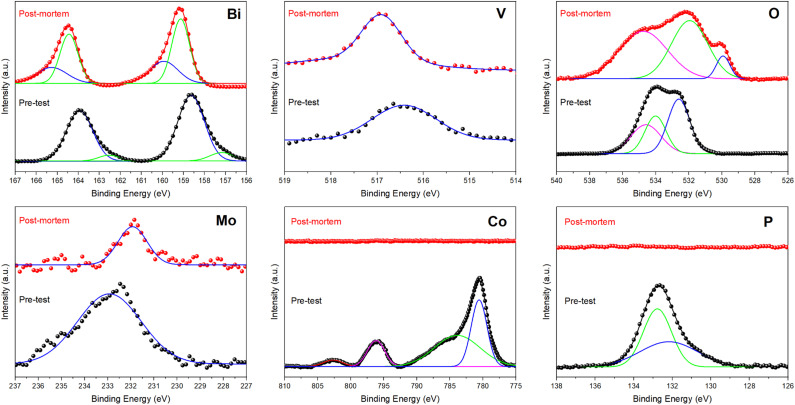
XPS spectra of pristine Mo:BiVO_4_ with CoPi on titanium felt (black dots, as in Fig. 3) and of Mo:BiVO_4_ with CoPi covered by a proton-exchange ionomer after the PEC tests in proton-exchange MPEA using liquid water at 30 °C for 15 hours (red dots), with the corresponding Lorentzian deconvolution for data fitting (lines) in the binding energy ranges for Bi, V, O, Mo, Co and P.

Fig. 7 compares the XPS spectra of the pristine Mo:BiVO_4_ samples with CoPi on Ti felt with those of the same samples covered by a proton-exchange ionomer which were used for the PEC tests with liquid water at 30 °C in the MPEA configuration. The two additional contributions to the Bi 4f peaks at 159.9 and 165.2 eV were attributed to Bi_2_O_3_, which may be a product of the photocorrosion reactions of BiVO_4_. This conclusion was also supported by the detection of additional features in the O 1 s spectrum, which shows a new peak at 529.9 eV attributed to Bi–O bonding and the formation of other oxides.^69^ The shift of the V peak from 516.4 eV to 517.6 eV possibly suggests no changes in the oxidation state of V(v), but a different surface interaction with the ionomer. The shift of the peak of Mo 3d_5/2_ to a lower binding energy at 231.9 eV suggests a variation of the oxidation state and the possible formation of different species, like molybdenum oxides.^70^ The most relevant effect of the PEC tests with liquid water at 30 °C was the total disappearance of Co 2p and P 2p signals, attributed to the complete co-catalyst dissolution.

**Fig. 8:**
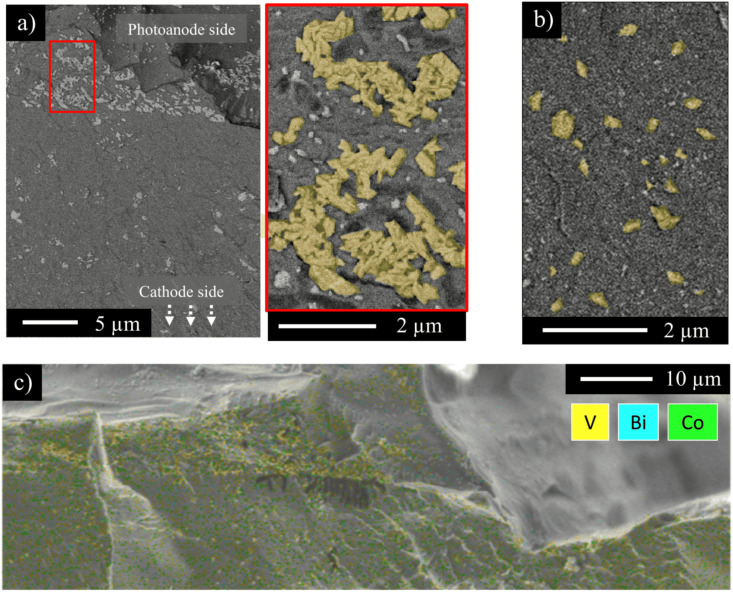
(a) and (b) False-colour backscattered electron SEM images of the cross-section of the proton-exchange membrane after the tests with liquid water at 56 °C: (a) close to the photoanode on Ti felt (cathode not reported) with a zoomed-in view of a detail in a red rectangle; (b) at the middle of the membrane thickness. (c) EDXS elemental mapping of the cross-section SEM image showing the solid particles formed in the proton-exchange membrane after the PEC tests with liquid water at 56 °C.

Examination of the false-colour cross-section SEM images of the proton-exchange membrane after the tests at 56 °C, shown in Fig. 8, reveals that solid particles were formed inside the membrane. These particles coalesced closer to the photoanode, forming clusters of few µm in size (Fig. 8(a)). Conversely, the characteristic dimension of the particles in the middle of the membrane was in the order of hundreds of nm (Fig. 8(b)). Analysing the EDXS elemental mapping, the solid particles were mainly composed of vanadium (Fig. 8(c)). From the Pourbaix diagram of V in water, solid V_2_O_5_ is predicted to be formed after the complete oxidation of vanadium at low pH and high concentration.^71,72^ The solubility of V_2_O_5_ is extremely low (0.7 mg L^−1^)^73^ and its supersaturation in the nanochannels of the proton-exchange membrane has likely occurred. Further research is necessary to define the exact mechanism behind the formation of these solid particles in the proton-exchange membrane of the MPEAs. Other solid species containing vanadium(iv) (*e.g.*, V_2_O_4_) or V(v) (*e.g.*, HVO_3_) could possibly have formed. The reactions to form V_2_O_5_ may have involved the dissolved oxygen or hydrogen in the membrane as already observed for the Pt band formation in proton exchange membrane fuel cells.^74^ The identification of the exact mechanism of this phenomenon is not in the scope of this work. Catalyst dissolution followed by the formation and coalescence of solid particles in the membrane has already been observed and modelled in PEM fuel cells^75–77^ and electrolyzers^78,79^ but it had never been reported for MPEAs.

### 3.5 Effects of photocorrosion in anion-exchange MPEAs after tests with liquid water

**Fig. 9:**
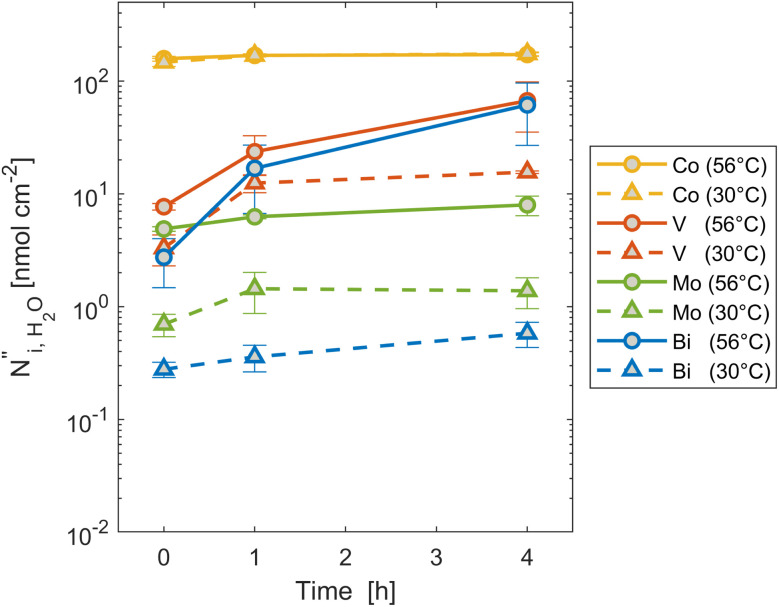
Moles of Bi, V, Mo or Co dissolved in liquid water per illuminated geometric area of the felt after chronoamperometry performed at different temperatures with anion-exchange MPEAs. The liquid samples were extracted after two potential–current density characteristic curves with simulated solar light.

As shown in Fig. 9, after operation of the anion-exchange MPEAs at 30 °C, the concentration of V in the anodic reservoir was more than one order of magnitude larger than that of Bi (
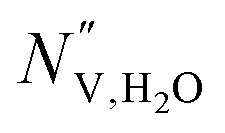
 (5 h, 30 °C) = 15.5 nmol cm^−2^*versus*
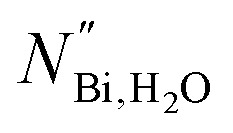
 (5 h, 30 °C) = 0.58 nmol cm^−2^). Furthermore, the amount of dissolved Bi detected was less than that of Mo (
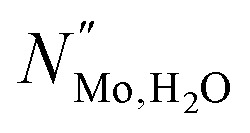
 (5 h, 30 °C) = 1.38 nmol cm^−2^). Operation at 56 °C accelerated the process of dissolution but it also modified the relative amounts of species found in liquid water, more closely following the theoretical stoichiometric ratios of the material. Toma *et al.*^31^ proposed that the self-passivation of BiVO_4_ in an alkaline environment with insoluble solid Bi oxides (Bi_4_O_7_ and Bi_2_O_3_) is kinetically inhibited despite the predictions from the thermodynamic first-principles calculations. The almost 1 : 1 molar ratio between bismuth and vanadium we obtained at 56 °C can confirm this theory but this also implies that temperature may have an impact on the mechanism of photocorrosion in an alkaline environment. Comparing these results with those of the chemical stability of the material, the applied potential and light have a more dominant effect than temperature in the dissolution of species into water (Fig. S19(d)–(f)). The accumulation of photogenerated holes at the surface of the photoanode in contact with the hydrated anion-exchange ionomer has a critical role in accelerating the photocorrosion reactions. The interpretation of the results is complicated because Bi, V and Mo are also in the stainless steel (SS) felt used as a substrate (Fig. S12). The chemical dissolution of species in anion-exchange MPEAs is lower than that of an SS felt coated with Mo:BiVO_4_ and CoPi (without an ionomer) left in a 0.01 M NaOH aqueous solution with pH ≈ 12 and even that of the bare SS felt after annealing (Fig. S20(b)). However, a dominance of V dissolution with respect to Bi and Mo could be observed in all three cases. During the OER in an alkaline environment, CoPi may experience a surface reconstruction which may affect its composition (towards the formation of Co oxides and hydroxides) and crystallinity.^80,81^ This reconstruction may explain the immediate dissolution of cobalt into the hydrated anion-exchange ionomer and its transfer to the anodic reservoir right after the two initial characteristic curves both at 30 and 56 °C (Fig. 9). Temperature appears not to have any relevant effect on this process in contrast to the application of an external potential and light, as shown by the assessment of the chemical stability of CoPi (Fig. S21).

**Fig. 10:**
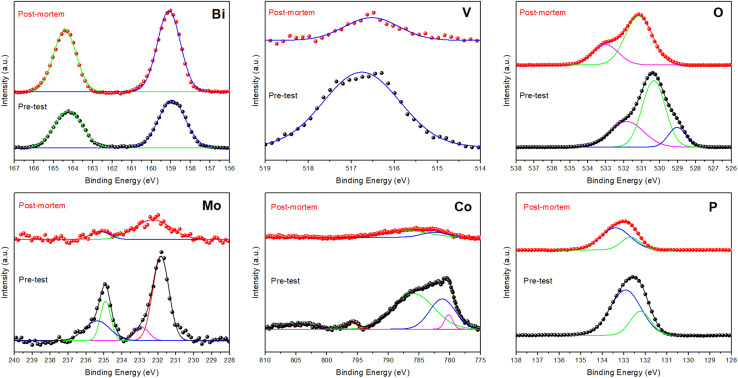
XPS spectra of pristine Mo:BiVO_4_ with CoPi on stainless steel felt (black dots) and of Mo:BiVO_4_ with CoPi covered by an anion-exchange ionomer after the PEC tests in anion-exchange MPEA using liquid water at 30 °C (red dots), with the corresponding Lorentzian deconvolution for data fitting (lines) in the binding energy ranges for Bi, V, O, Mo, Co and P.

Fig. 10 compares the XPS spectra of pristine Mo:BiVO_4_ samples with CoPi on SS felt with those of the same ones covered by an anion-exchange ionomer after the PEC tests with liquid water at 30 °C in the MPEA configuration. No relevant variations of the oxidation states of the different elements were observed, despite the complications in the analysis of the results caused by the elements which are in the SS felt (Fig. S12). However, the primary effect of PEC tests can be attributed to the almost complete loss of Co 2p signals due to co-catalyst dissolution.

The false-colour SEM images of the cross section of the anion-exchange membrane used for the tests at 30 °C show the formation of agglomerates in the order of 30 nm homogeneously distributed along its thickness (Fig. S22). These agglomerates were only composed of C and Br, the main constituents of the Fumasep™ membrane, and could therefore not be associated with the photoanode dissolved species (Fig. S23).

### 3.6 PEC characterization with water vapour

**Fig. 11:**
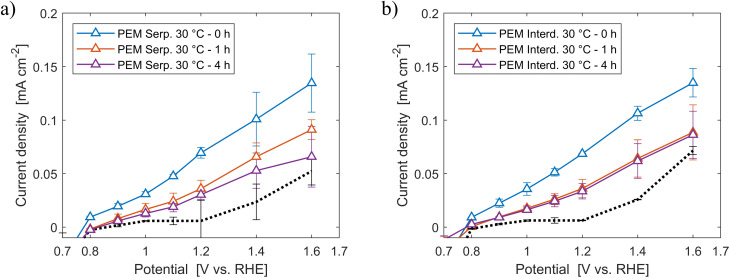
(a) and (b) Current density–potential characteristic curves of pristine proton-exchange MPEAs after different time spans of chronoamperometry at 1.23 V *vs.* RHE with water in the gas phase at 30 °C using (a) serpentine flow field geometry or (b) interdigitated flow field geometry. The black dotted lines in the figure represent the characteristic curves with water in the gas phase of the pristine MPEAs in the dark.

Due to the inferior performance in liquid-phase water experiments of anion-exchange MPEAs compared to proton-exchange MPEAs, gas-phase tests were exclusively conducted with the latter ones. Operation with water in the gas phase resulted in comparable onset potentials to the ones obtained using liquid-phase water but a lower current density (approximately 85% lower at 1 V *vs.* RHE) (Fig. 11(a) and (b)). This behaviour is attributed to the fact that gas-phase operation results in a worse hydration of the proton-exchange ionomer, reducing conductivity, and the reduced availability of water at the photoanode–electrolyte interface. The reduced membrane hydration also leads to a more acidic pH inside the nanochannels of the ionomer, potentially impacting the electrochemical reaction. The performance of pristine proton-exchange MPEAs with water vapour was unaffected by the flow field geometry or the imposed volumetric flow rate, which was varied from 20 to 220 mL min^−1^. This suggests that water transport from the channels of the photo-electrolyzer to the active sites was not the limiting factor at these values of current density. Consequently, the geometry that minimizes the pressure gradient across the cell should be favoured. The photocorrosion of the material may be the cause of a faster reduction of the current density in time with respect to that observed with liquid water (Fig. S24). The lower local pH may induce more detrimental operating conditions, leading to faster photocorrosion and dissolution of the material.

## 4 Conclusions

The performance and the stability of membrane-photoelectrode assemblies with Mo-doped BiVO_4_ photoanodes on metallic felt with CoPi co-catalysts were assessed for the first time using water in the liquid and gas phase at different temperatures.

The optimal proton-exchange MPEAs tested with liquid water at different temperatures showed an increase in the onset potential with temperature from 0.77 V *vs.* RHE at 30 °C to 0.83 V *vs.* RHE at 56 °C, possibly due to a more pronounced surface recombination rate of photogenerated holes compared to their transfer to the electrolyte. The increase in saturation current density with temperature from 0.30 mA cm^−2^ at 30 °C to 0.40 mA cm^−2^ at 56 °C was explained by a decreased bandgap and increased absorption coefficients resulting in more light absorption and by the better reaction kinetics. The reduction of the saturation current density over time is attributed to the photocorrosion of the photoanode, which reduced the amount of photoactive material over time, thereby reducing the amount of absorbed light. The photocorrosion of the photoanode led to its dissolution, but this was more pronounced for V than Bi. The dissolved vanadium also formed solid particles in the proton-exchange membrane with diameters in the order of hundreds of nm, which coalesced in larger agglomerates of few µm in the region closer to the anodic side. The CoPi co-catalyst chemically dissolved due to the local acidic pH imposed by the proton-exchange ionomer.

With anion-exchange MPEAs, the saturation photocurrent density only reached 0.1 mA cm^−2^ at 30 °C. It decreased to 0.05 mA cm^−2^ at 56 °C. These reductions may be attributed to a less efficient charge extraction at the photoanode–electrolyte junction than with the proton-exchange ionomer. The photocorrosion reactions caused the dissolution of Bi, V and Mo. The CoPi co-catalyst was completely dissolved after the first two potential–current density characteristic curves.

The performance of proton-exchange MPEAs with vapour was characterized at lower current densities with a reduction of 85% at 1 V *vs.* RHE and 30 °C, likely due to the worse hydration of the solid ionomer. Operation with vapour resulted in a faster decrease in the saturation current density during PEC tests than with liquid-phase water. The more acidic pH at the interface with the solid electrolyte possibly led to a faster photocorrosion and dissolution of the photoanode.

The deposition of solid ionomers onto the semiconductor enables operation of membrane-photoelectrode assemblies with liquid water and humid air at the expense of performance losses. Photocorrosion penalizes the operation of these devices and additional research work is required to identify the right combination of semiconductors and co-catalysts in order to reach the ambitious targets of durability required for the large-scale implementation of photoelectrochemical water splitting.

## Author contributions

R.V.: conceptualization, methodology, investigation, formal analysis, data curation, writing – original draft; S.G.: conceptualization, methodology, investigation, formal analysis, data curation, writing – review & editing; I.H.-G. and F.S.: conceptualization, methodology, writing – review & editing; E.C.C.: funding acquisition, supervision, writing – review & editing and S.H.: funding acquisition, conceptualization, supervision, project administration, writing – review & editing.

## Conflicts of interest

There are no conflicts of interest to declare.

## Supplementary Material

SE-010-D6SE00417B-s001

## Data Availability

The data supporting this article has been included as part of the supplementary information (SI). Supplementary information is available. See DOI: https://doi.org/10.1039/d6se00417b.
