# Supplementary material for: Performance and stability of membrane-photoelectrode assemblies with BiVO4 photoanodes for water splitting
Source: Sustain Energy Fuels. 2026 Jun 19;10(15):3669–83. doi: 10.1039/d6se00417b (PMC13334422; doi:10.1039/d6se00417b)
Supplement: SE-010-D6SE00417B-s001 [file SE-010-D6SE00417B-s001.pdf]

Supplementary Information (SI) for Sustainable Energy & Fuels.
This journal is © The Royal Society of Chemistry 2026

Supporting Information

Performance and stability of membrane-photoelectrode assemblies with $BiVO_4$ photoanodes for water splitting

Roberto Valenza,[a] Sebastiano Gadolini,[b] Isaac Holmes-Gentle,[a] Francesco Spanu,[b] Elena C. Corbos[b] and Sophia Haussener[a,*]

[a] *Laboratory of Renewable Energy Science and Engineering, École Polytechnique Fédérale de Lausanne, 1015 Lausanne, Switzerland*

[b] *Johnson Matthey Technology Centre, Blounts Court Road, Reading RG4 9NH, United Kingdom*

[*] Corresponding author: sophia.haussener@epfl.ch

Contents

S1 Supplementary methods **2**

S2 Supplementary results **4**

S1. Supplementary methods

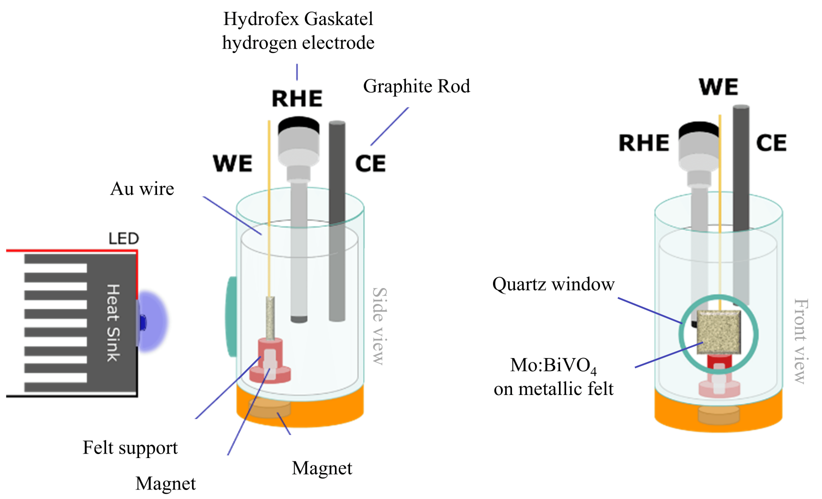

Figure S1: Simplified schematics of the side view (left) and of the front view (right) of the three-electrode setup used for the CoPi co-catalyst photoelectrodeposition and for the preliminary PEC tests in 0.2 M Na_2SO_4 aqueous solution for the optimization of the synthesis of the Mo:$BiVO_4$ photoanodes on metallic felts.

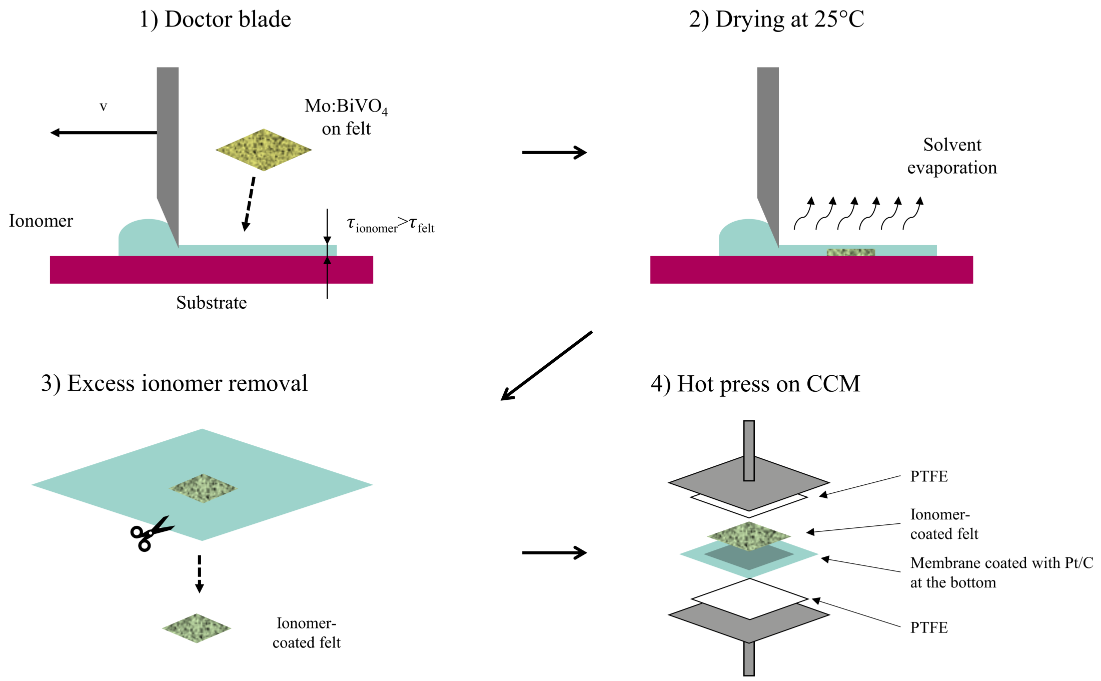

Figure S2: Simplified schematics of the different phases to prepare the membrane-photoelectrode assemblies: 1) doctor blade coating of the Mo:$BiVO_4$ photoanodes on metallic felts with ionomer; 2) drying at 25 °C to ensure the solvent evaporation; 3) removal of the excess ionomer with a scissor; 4) hot press of the Mo:$BiVO_4$ photoanodes on metallic felts coated with ionomer on a catalyst-coated membrane (CCM) with a Pt/C cathode catalyst layer.

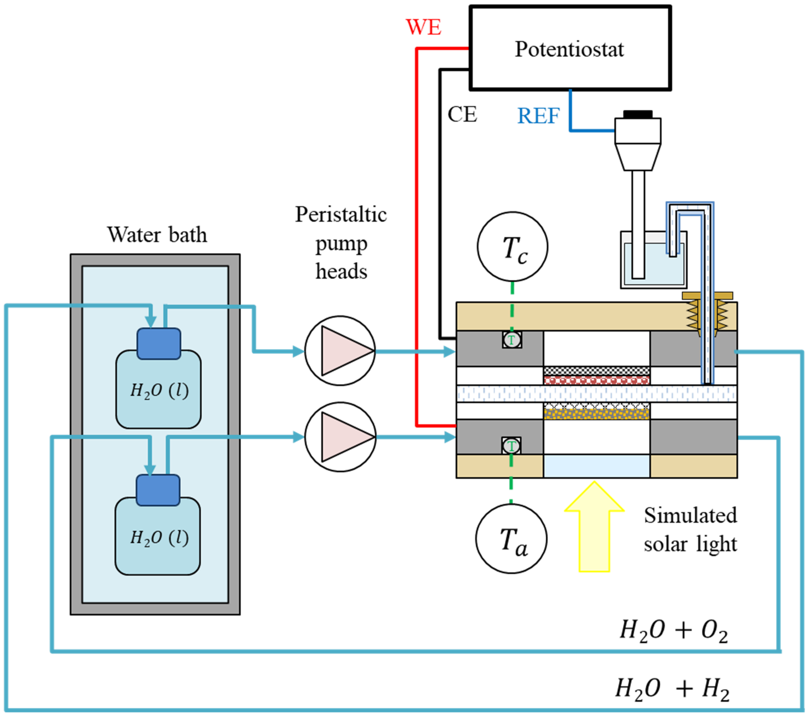

Figure S3: Simplified schematic of the setup used to perform tests with the photo-electrolyzer using water in liquid phase as reactant.

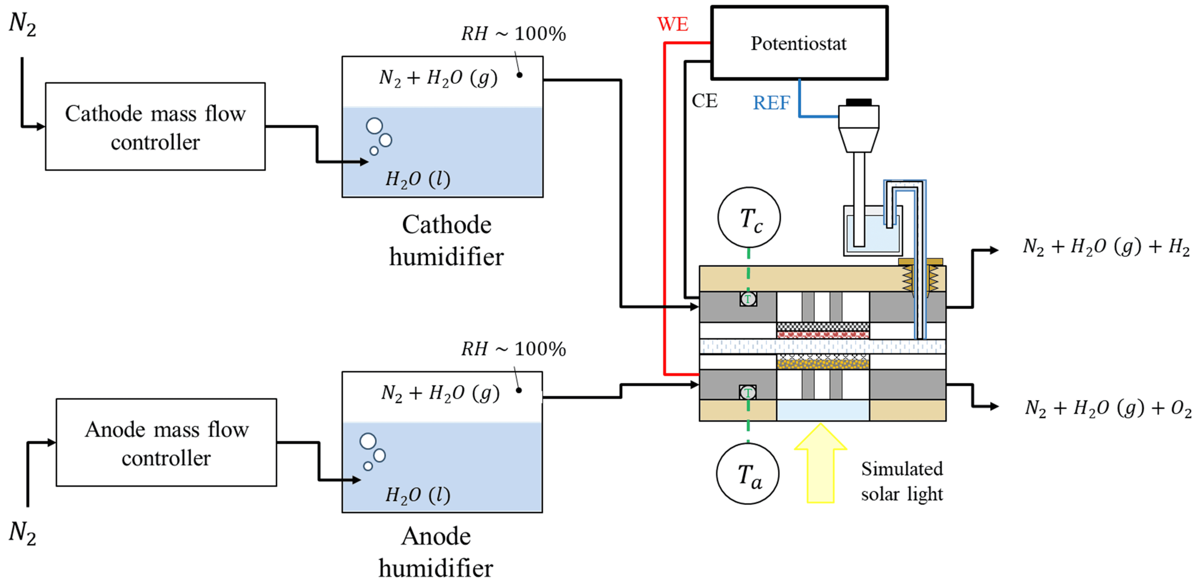

Figure S4: Simplified schematic of the setup used to perform tests with the photo-electrolyzer using water in vapour phase as reactant.

S2. Supplementary results

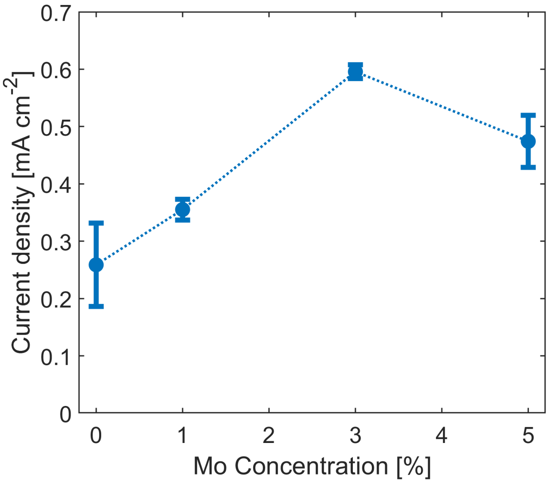

Figure S5: Current density at 1.23 V *vs.*. RHE during the third forward sweep of the cyclic voltammetries in 0.2 M Na_2SO_4 aqueous solution of the $BiVO_4$ photoanodes deposited on Ti felts with 8 s-SILAR cycles as a function of the Mo dopant molar concentration.

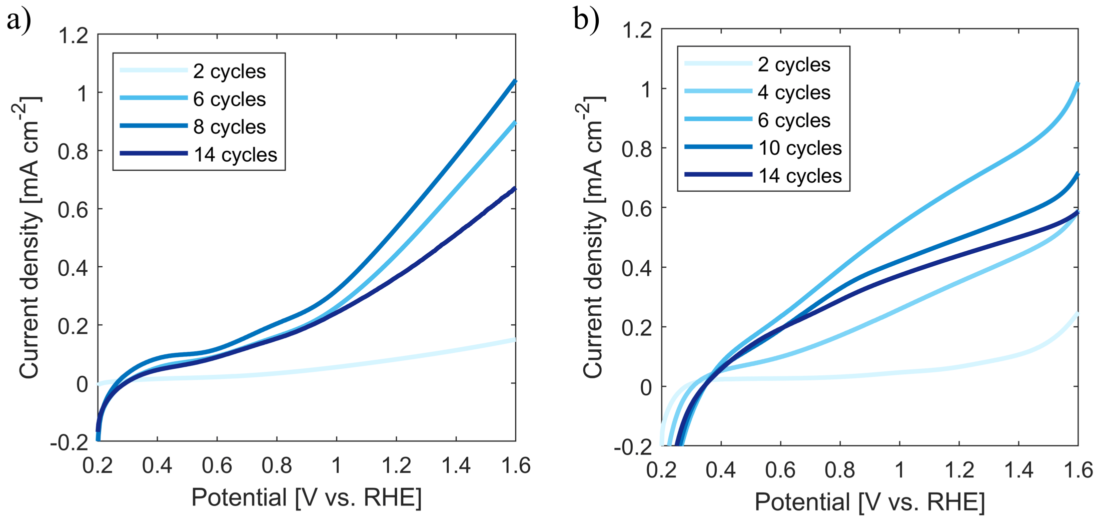

Figure S6: Current density as a function of the applied potential during the third forward sweep of the cyclic voltammetries in 0.2 M Na_2SO_4 aqueous solution of $BiVO_4$ with 3% Mo doping photoanodes deposited with a different number of s-SILAR cycles: (a) on Ti felts; (b) on stainless steel felts.

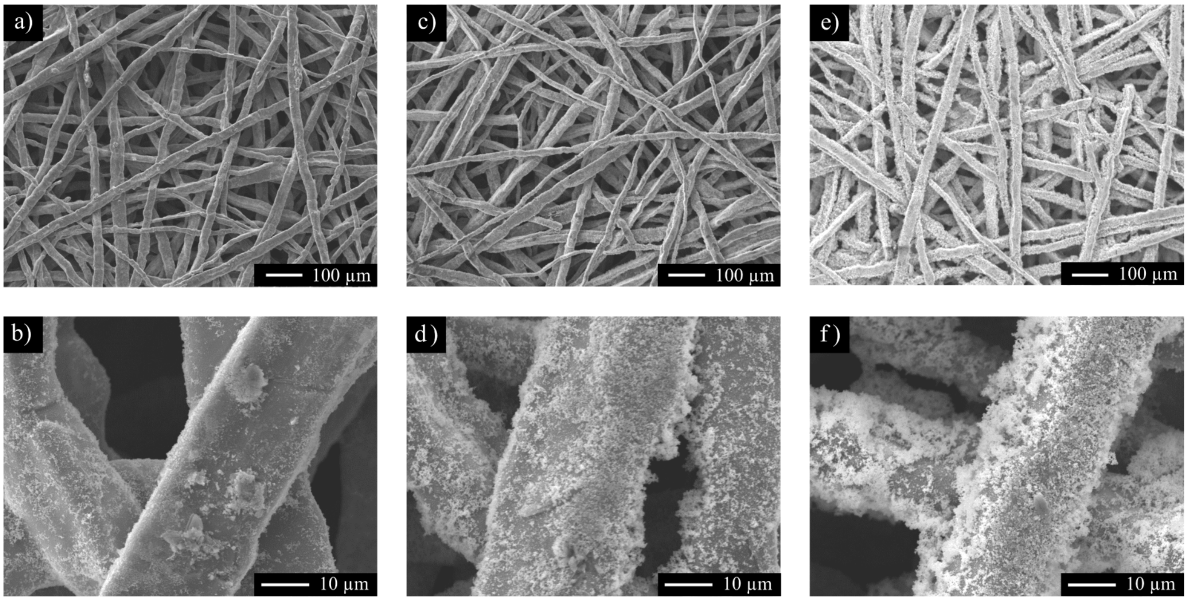

Figure S7: SEM images of the Ti felts coated with Mo:$BiVO_4$ after a different number of s-SILAR cycles: (a)-(b) 2 cycles; (c)-(d) 8 cycles; (e)-(f) 14 cycles.

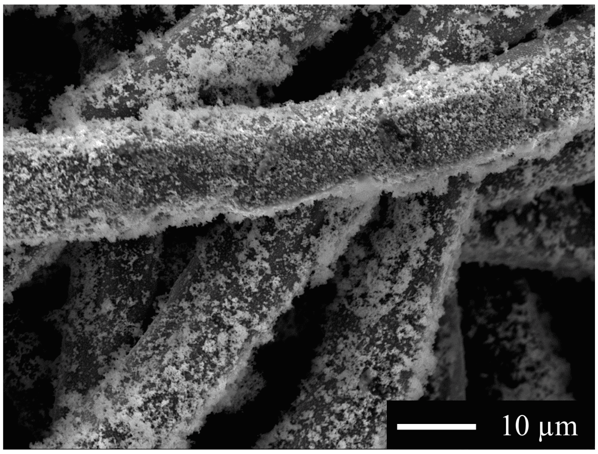

Figure S8: SEM images of the fibres of the SS felts coated with Mo:$BiVO_4$ after 6 s-SILAR cycles.

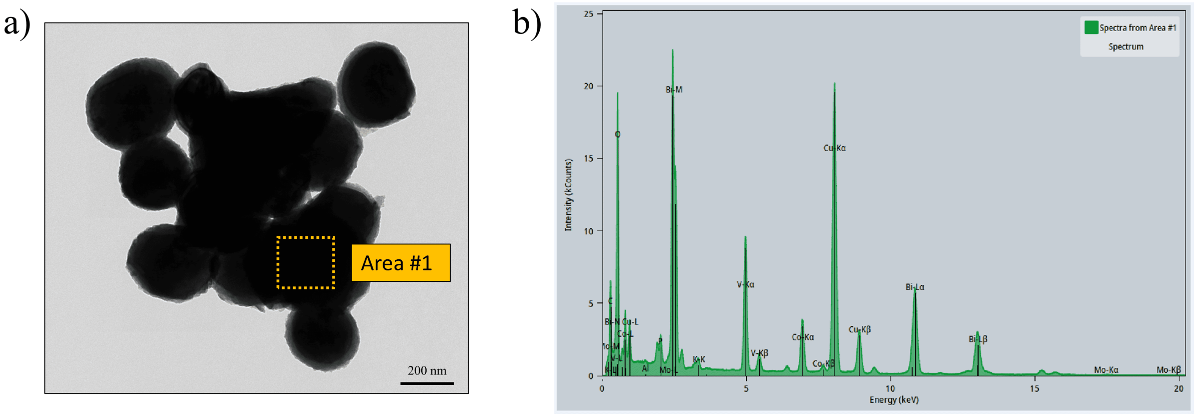

Figure S9: (a) Energy-dispersive X-ray spectroscopy (EDXS) elemental mapping of a Mo:$BiVO_4$ nanostructure with CoPi co-catalyst with highlight of the area considered for the spectrum; (b) EDXS spectrum of the highlighted area.

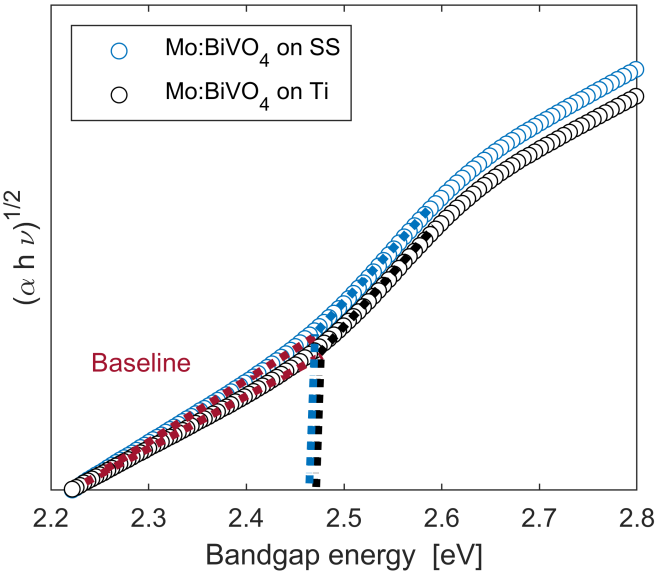

Figure S10: Tauc's plot of Mo:$BiVO_4$ photoanodes on titanium and stainless steel felts with linear fitting of the band dispersion corrected for the baseline as previously proposed [1]. The density of ionized donors and the flatband potential was not estimated due to the nanostructured morphology of the photoanode [2, 3]

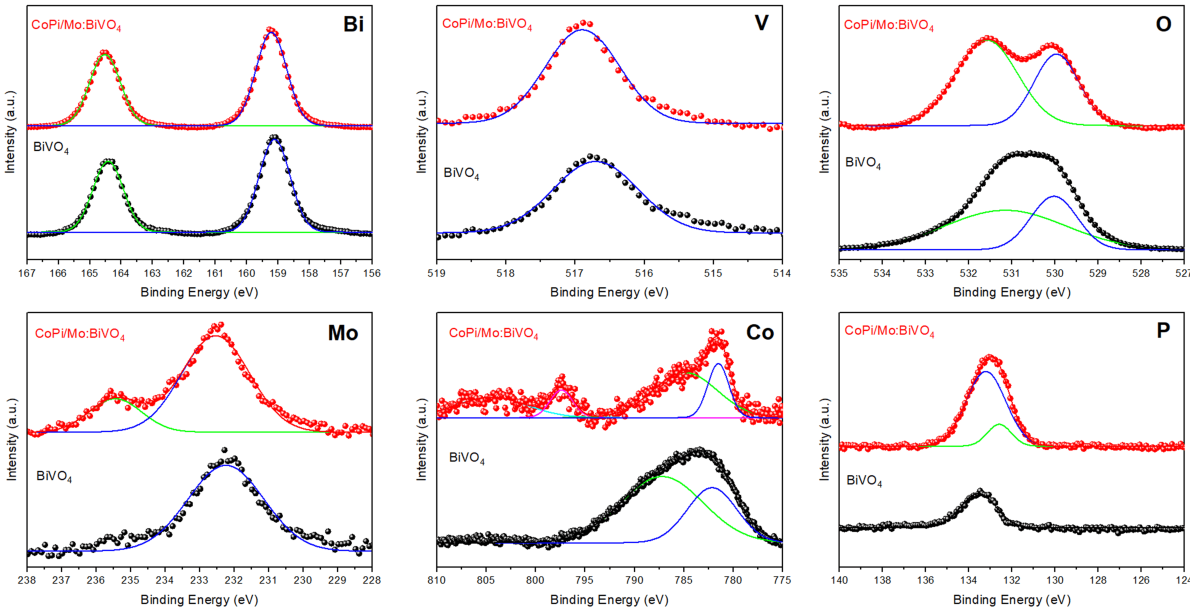

Figure S11: XPS spectra of pristine $BiVO_4$ (black dots) and Mo:$BiVO_4$ with CoPi (red dots) on stainless steel felt with the corresponding Lorentzian deconvolution for data fitting (lines) in the binding energy ranges for Bi, V, O, Mo, Co and P.

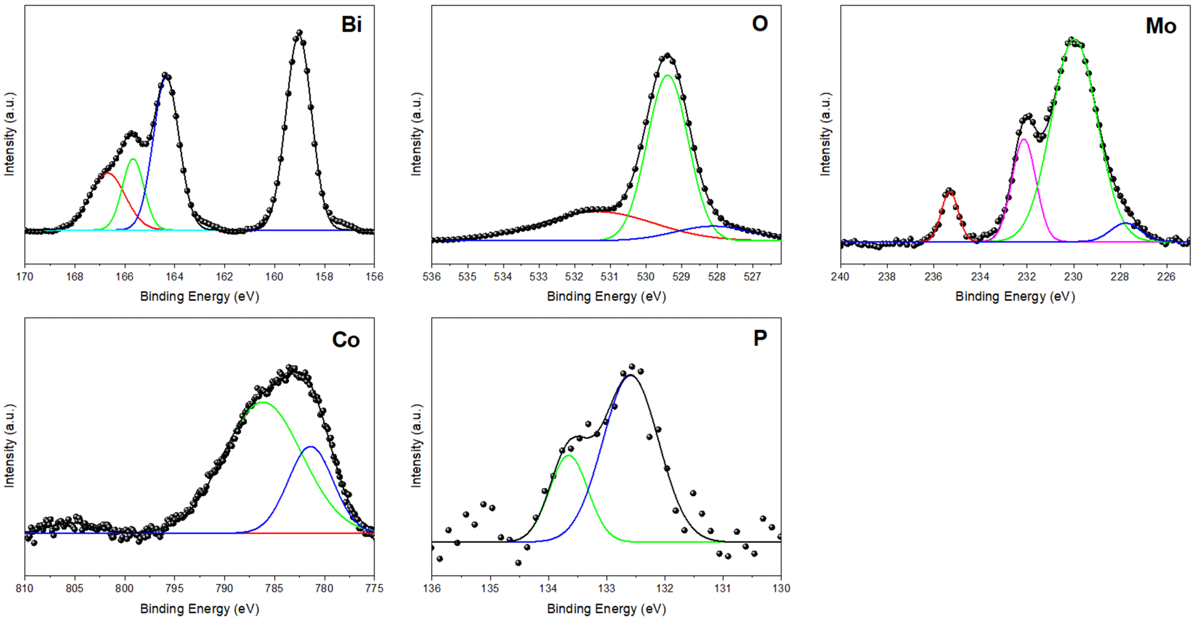

Figure S12: XPS spectra of the bare stainless steel felt after annealing (black dots) with the corresponding Lorentzian deconvolution for data fitting (lines) in the binding energy ranges for Bi, O, Mo, Co and P.

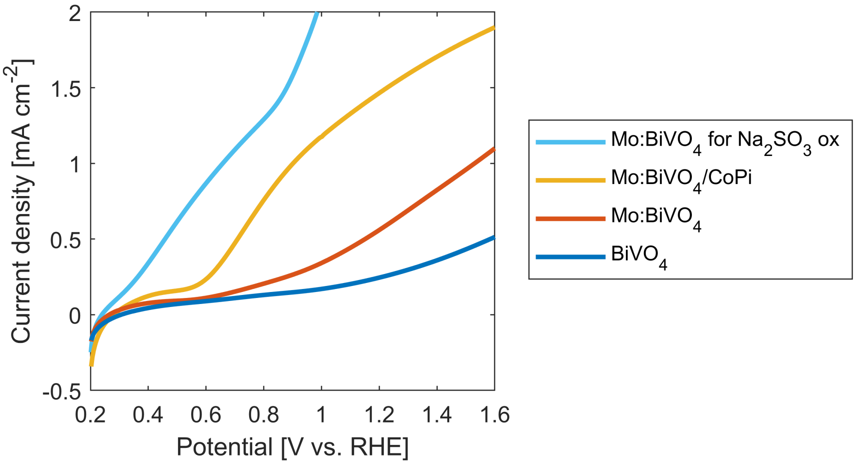

Figure S13: Current density as a function of the applied potential during the third forward sweep of the cyclic voltammetries in 0.2 M Na_2SO_4 aqueous solution of different samples on Ti felts: Mo:$BiVO_4$ with CoPi for Na_2SO_3 oxidation (0.1 M Na_2SO_3 was added to the electrolyte), Mo:$BiVO_4$ with CoPi, Mo:$BiVO_4$ and $BiVO_4$, all exposed to blue light. In dark, the oxidation of Na_2SO_3 determined a dark current density with an onset potential at approximately 0.9 V *vs.* RHE which did not allow to calculate the catalytic efficiency η_{cat} for larger potentials.

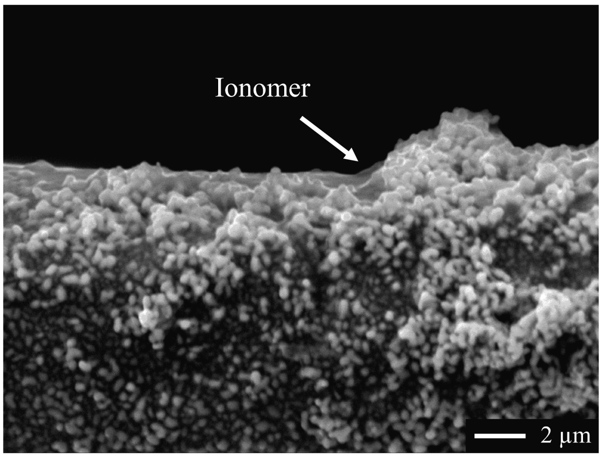

Figure S14: Mo:$BiVO_4$ with CoPi on SS fibre after anion-exchange ionomer deposition.

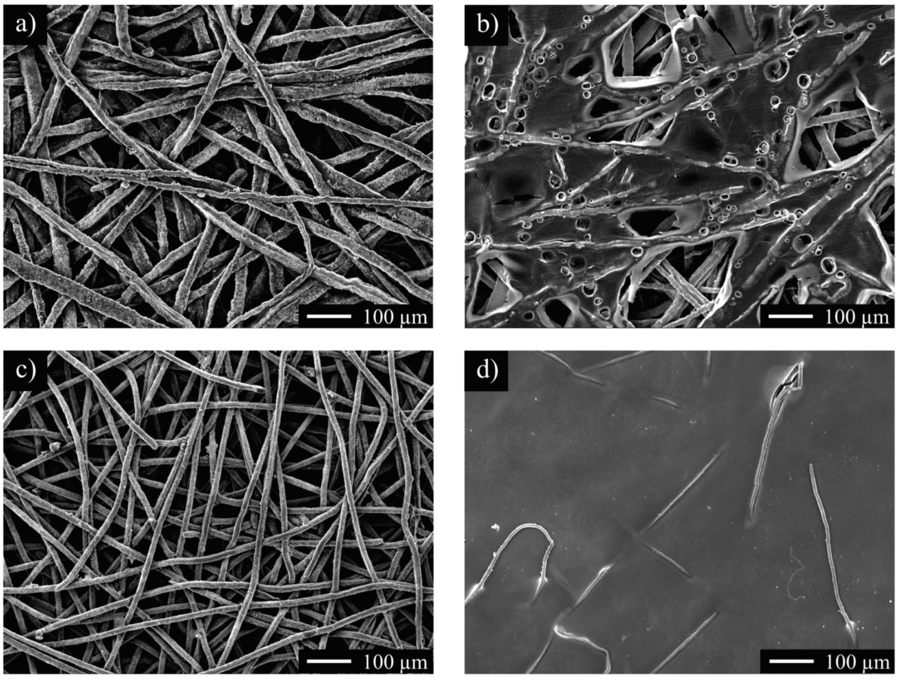

Figure S15: (a)-(b) Mo:$BiVO_4$ with CoPi on Ti felt after proton-exchange ionomer deposition: (a) side directly exposed to light, (b) side in contact with the membrane during hot pressing. (c)-(d) Mo:$BiVO_4$ with CoPi on SS felt after anion-exchange ionomer deposition: (c) side directly exposed to light, (d) side in contact with the membrane during hot pressing.

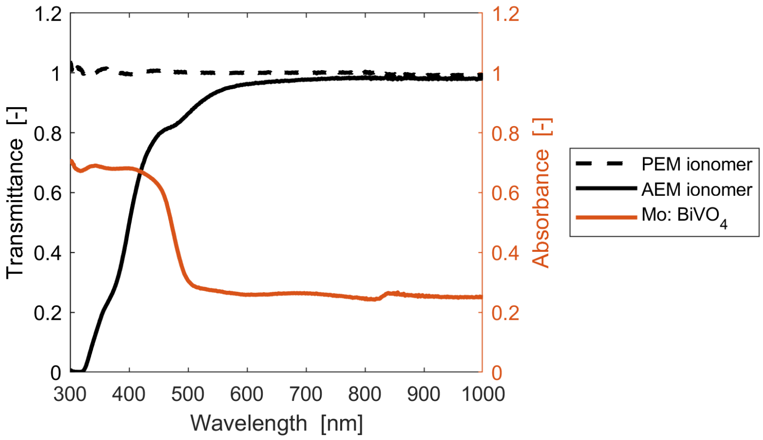

Figure S16: Transmittance of a 10 μm layer of proton-exchange ionomer (dashed line) and of a 60 μm layer of anion-exchange ionomer (solid line) compared to the absorbance spectrum of a Mo:$BiVO_4$ photoanode on a planar FTO-coated glass substrate (in orange, right axis). The thicknesses of the two ionomer layers do not represent the ones observed on the fibres but they were chosen to determine qualitative transmittance results.

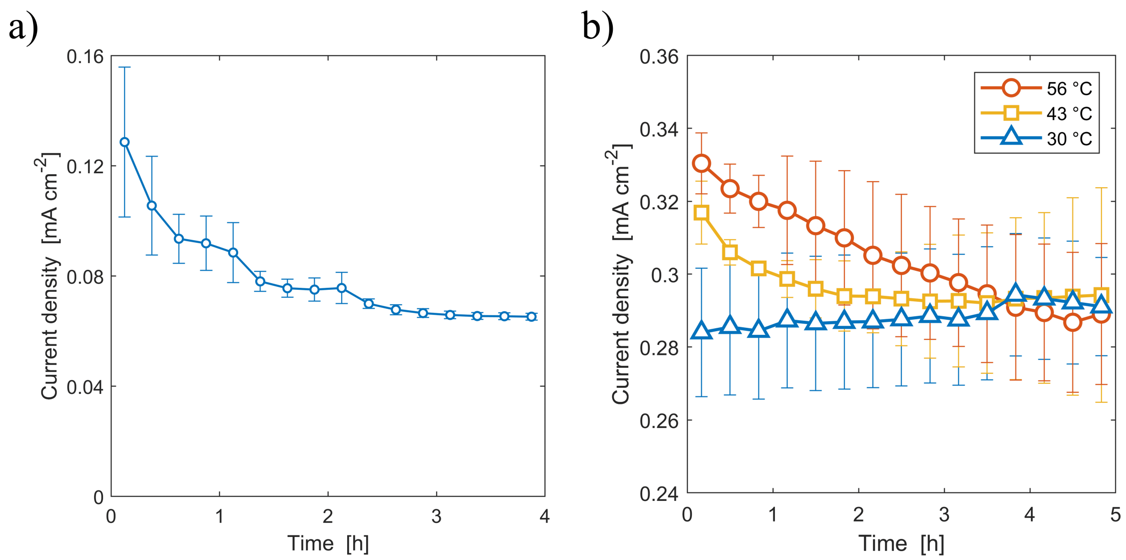

Figure S17: (a) Moving averages of the current density measured during the chronoamperometry at 1.23 V *vs.* RHE and 30 °C with anion-exchange MPEAs. (b) Moving averages of the current density measured during the first five hours of the chronoamperometry at 1.23 V *vs.* RHE with proton-exchange MPEAs tested at different temperatures.

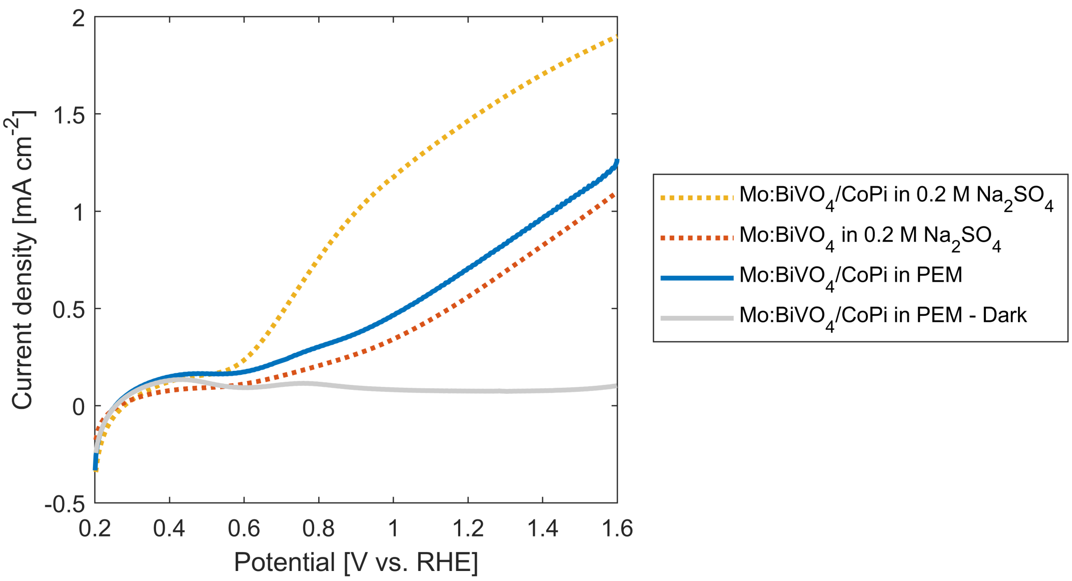

Figure S18: Current density as a function of the applied potential during the third forward sweep of the cyclic voltammetries with blue LED light or dark of the pristine proton-exchange MPEAs with Mo:$BiVO_4$ and CoPi using liquid water compared to the ones of Mo:$BiVO_4$ on Ti felt with and without CoPi in 0.2 M Na_2SO_4 aqueous solution.

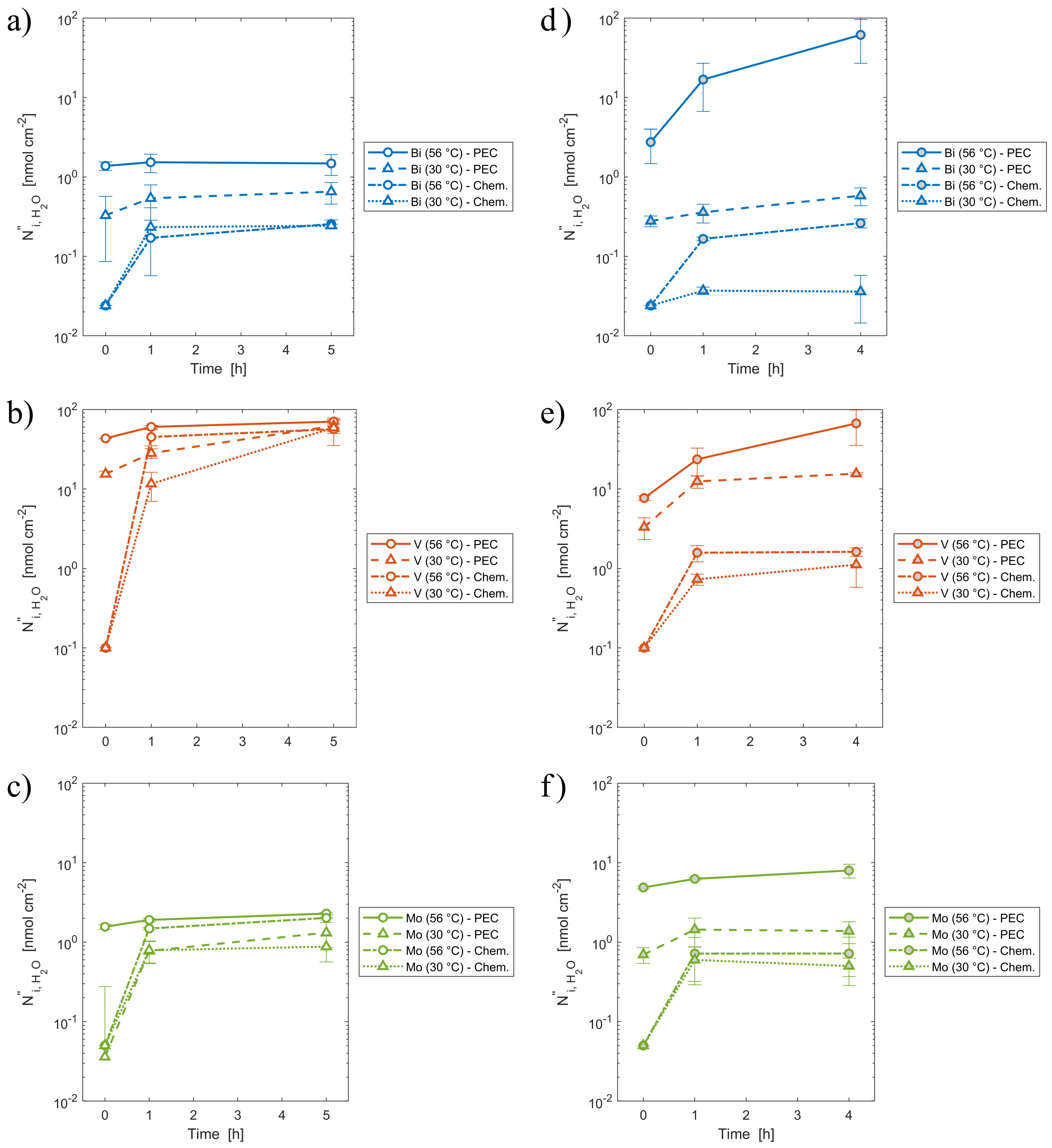

Figure S19: (a)-(b)-(c) Moles of (a) Bi, (b) V or (c) Mo dissolved in water per illuminated geometric area of the felts after the photoelectrochemical or chemical stability tests performed at different temperatures with proton-exchange MPEAs using liquid water. (d)-(e)-(f) Moles of (d) Bi, (e) V or (f) Mo dissolved in water per illuminated geometric area of the felts after the photoelectrochemical or chemical stability tests performed at different temperatures with anion-exchange MPEAs using liquid water. For the PEC stability tests, the liquid samples were extracted after two potential-current density characteristic curves with simulated solar light.

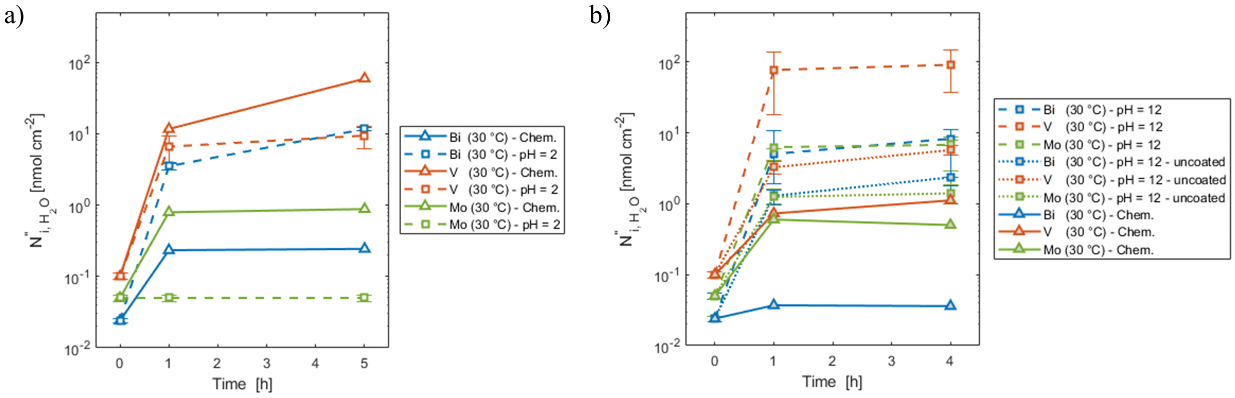

Figure S20: (a) Moles of Bi, V or Mo dissolved in solution per illuminated geometric area of the felts after the chemical stability tests with proton-exchange MPEAs (*i.e.*, Mo:$BiVO_4$/CoPi on Ti felts integrated in PEM assemblies) in liquid water (triangles) or with the Ti felt coated with Mo:$BiVO_4$ and CoPi (without ionomer) in a 0.005 M H_2SO_4 aqueous solution with pH≈2 (squares). (b) Moles of Bi, V or Mo dissolved in solution per illuminated geometric area of the felts after the chemical stability tests with anion-exchange MPEAs (*i.e.*, Mo:$BiVO_4$/CoPi on SS felts integrated in AEM assemblies) in liquid water (triangles) or with the SS felt either uncoated or coated with Mo:$BiVO_4$ and CoPi (without ionomer) in a 0.01 M NaOH aqueous solution with pH≈12 (squares).

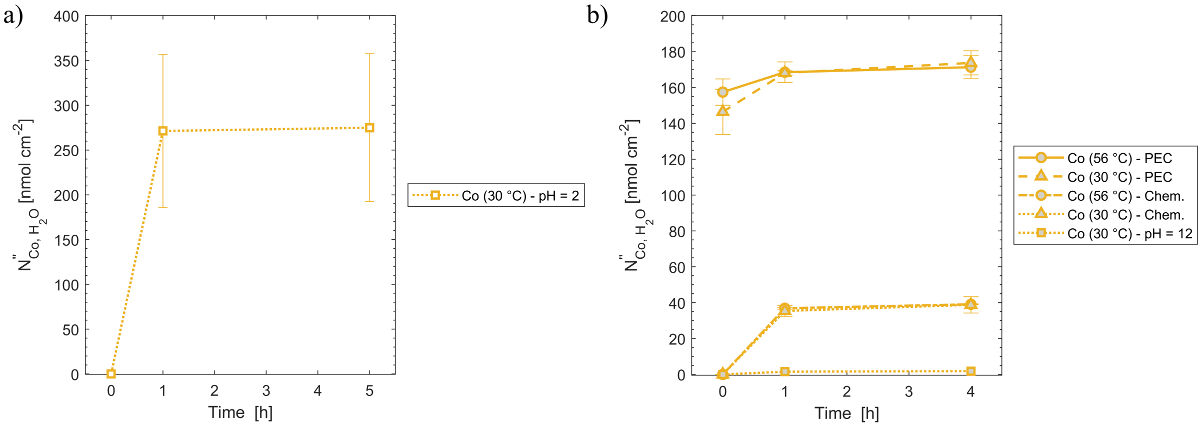

Figure S21: (a) Moles of Co dissolved in solution per illuminated geometric area of the felts after the chemical stability tests with the Ti felt coated with Mo:$BiVO_4$ and CoPi (without ionomer) in a 0.005 M H_2SO_4 aqueous solution with pH≈2. (b) Moles of Co dissolved in solution per illuminated geometric area of the felts after the photoelectrochemical or chemical stability tests with anion-exchange MPEAs in liquid water at different temperature (circles for 56 °C or triangles for 30 °C) or with the SS felt coated with Mo:$BiVO_4$ and CoPi (without ionomer) in a 0.01 M NaOH aqueous solution with pH≈12 (squares). For the PEC stability tests, the liquid samples were extracted after two potential-current density characteristic curves with simulated solar light.

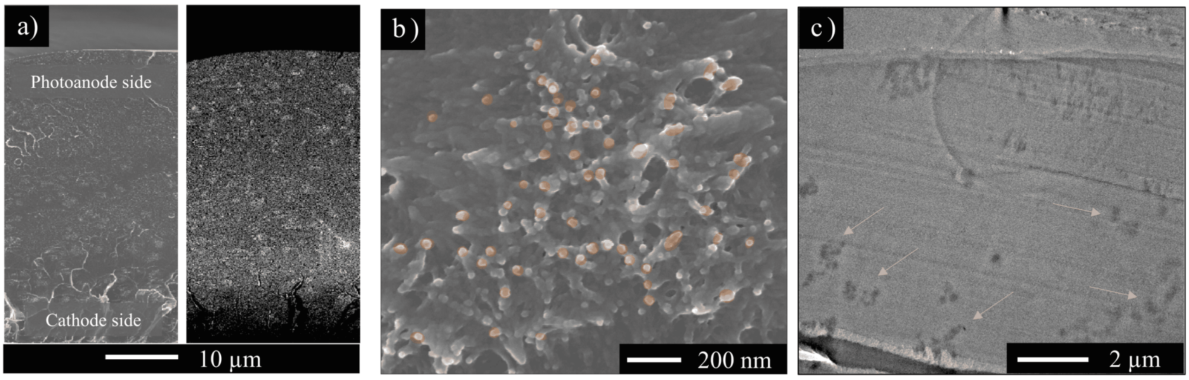

Figure S22: (a)-(b) SEM images of the cross-section of the anion-exchange membrane after the tests with liquid water at 30 °C: (a) of the membrane next to the high-contrast backscattered electron SEM image of the same cross-section; (b) of a detail of the membrane with false-colour highlights of the observed agglomerates. (c) TEM images of the same cross section highlighting the same agglomerates.

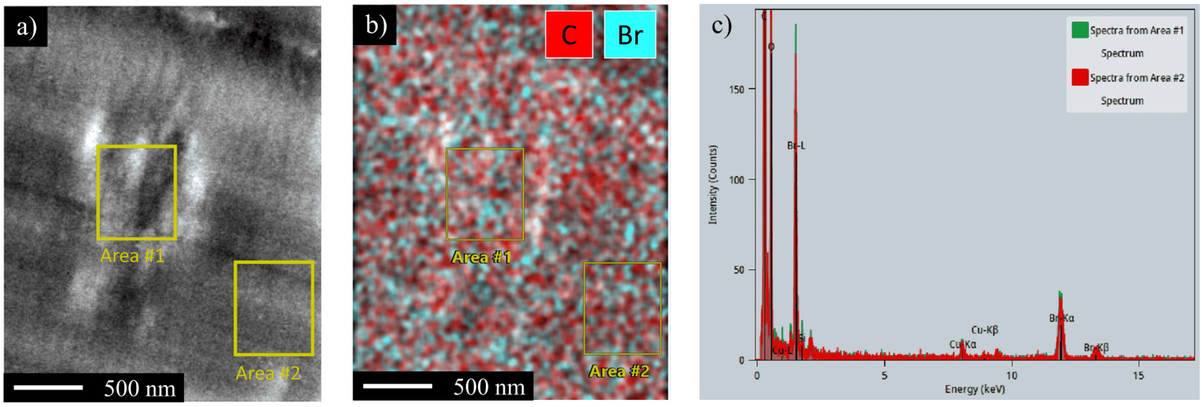

Figure S23: (a) Scanning transmission electron microscopy (STEM) image of one of the agglomerates observed in the aged anion-exchange membrane cross-section. (b) EDXS spatial elemental mapping of C and Br in the same STEM image. (c) EDXS spectra of the two areas highlighted in (a) and (b).

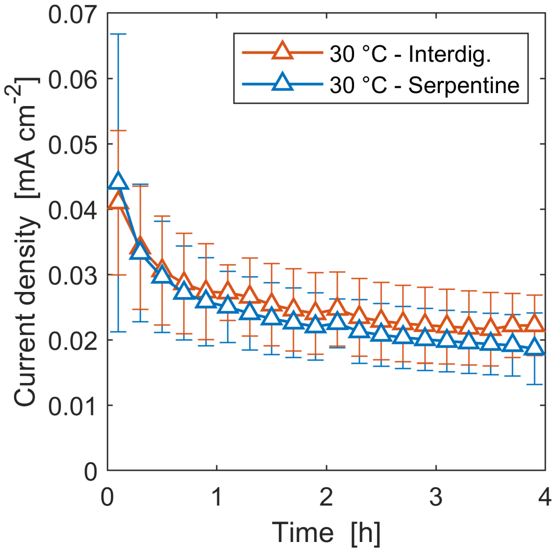

Figure S24: Moving averages of the current density measured during the chronoamperometry at 1.23 V *vs.* RHE and 30 °C with proton-exchange MPEAs tested using water in gas phase with serpentine or interdigitated flow field plates.

Table S1: Summary of previous work on membrane photoelectrode assemblies: photoelectrodes, operating conditions, membrane systems and photocurrent values. The table specifies if the study focuses on the photocorrosion of the electrodes, the effects of temperature or if it proposes complex flow field geometries.

Photoelectrode	Operating conditions	Membrane system	Photocurrent	Analysis of photocorrosion	Analysis effects of temperature	Study of a complex flow field	References
Ti/TiO_2	vapour-fed (RH = 60%)<br>aqueous phase (0.1 M Na_2SO_4)	proton-exchange, three-electrode system	0.43 mA cm^{-2} at 1.23 V *vs.* RHE<br>0.65 mA cm^{-2} at 1.23 V *vs.* RHE	No	No	No	[4]
Ti/W/$BiVO_4$	vapour-fed (RH = 60%)<br>aqueous phase (0.1 M Na_2SO_4)	proton-exchange, three-electrode system	1.55 mA cm^{-2} at 1.23 V *vs.* RHE<br>2.1 mA cm^{-2} at 1.23 V *vs.* RHE	No	No	No	[5]
Ti/WO_3/Mo/$BiVO_4$	vapour-fed (RH > 90%)<br>aqueous phase (Water)	proton-exchange, three-electrode system	0.74 mA cm^{-2} at 1.23 V *vs.*. RHE<br>0.57 mA cm^{-2} at 1.23 V *vs.* RHE	No	No	No	[6]
Ti/$SrTiO_3$	vapour-fed (RH = 60%)<br>aqueous phase (Water)	proton-exchange, three-electrode system	0.3 mA cm^{-2} at 0.3 V *vs.* RHE<br>0.45 mA cm^{-2} at 0.3 V *vs.* RHE	No	No	No	[7]
Ti/$SrTiO_3$	vapour-fed (RH = 95%)	proton-exchange, two-electrode system	2.5 mA cm^{-2} at 1.2 V *vs.* RHE	No	Yes	No	[8]
Carbon/TiO_2	vapour-fed (RH = 60%)	proton-exchange, two-electrode system	27 μA cm^{-2} at 0 V	No	No	No	[9]
Carbon/TiO_2	aqueous phase (water)	proton-exchange, two-electrode system	47 μA cm^{-2} at 0 V	No	No	No	[10]
Ti/TiO_2 nanotubes	vapour-fed (3% H_2O in Ar)	proton-exchange, three-electrode system	2 mA cm^{-2} at 1.2 V *vs.* RHE	No	No	No	[11]
TPCS[a]/CuSCN/BHJ[b]	vapour fed (humid Ar gas)	proton-exchange, three-electrode system	1.1 mA cm^{-2} at 0 V *vs.* RHE	No	No	No	[12]

Continued on next page

Photoelectrode	Operating conditions	Membrane system	Photocurrent	Analysis of photocorrosion	Analysis effects of temperature	Study of a complex flow field	References
Ti/α-Fe_2O_3	aqueous phase (1 M NaOH)	anion-exchange, three-electrode system	0.52 mA cm^{-2} at 1.23 V *vs.* RHE	No	No	No	[13]
Ti/W/$BiVO_4$-B/NiFeOOH	aqueous phase (0.5 M KBi)	anion-exchange, three-electrode system	3.5 mA cm^{-2} at 1.23 V *vs.* RHE	No	No	No	[14]
Ti/Mo/$BiVO_4$/$NiFeO_x$	vapour-fed	anion-exchange, two-electrode system	0.9 mA cm^{-2} at 1.2 V	Yes	No	No	[15]
	aqueous phase (0.5 M KBi)		1.74 mA cm^{-2} at 1.2 V				
Ti/Mo:$BiVO_4$/CoPi	aqueous phase (water)	proton-exchange, three-electrode system	0.30 mA cm^{-2} at 1.23 V *vs.* RHE	Yes	Yes	Yes	This work
	vapour-fed	proton-exchange, three-electrode system	0.045 mA cm^{-2} at 1.23 V *vs.* RHE				
SS/Mo:$BiVO_4$/CoPi	aqueous phase (water)	anion-exchange, three-electrode system	0.10 mA cm^{-2} at 1.23 V *vs.* RHE	Yes	Yes	No	This work

[a]TPCS = transparent porous conductive substrate. [b]BHJ = bulk heterojunction.

References

[1] P. Makuła, M. Pacia and W. Macyk, *The Journal of Physical Chemistry Letters*, 2018, **9**, 6814–6817.

[2] K. Sivula, *ACS Energy Letters*, 2021, **6**, 2549–2551.

[3] A. Hankin, F. E. Bedoya-Lora, J. C. Alexander, A. Regoutz and G. H. Kelsall, *Journal of Materials Chemistry A*, 2019, **7**, 26162–26176.

[4] G. Zafeiropoulos, H. Johnson, S. Kinge, M. C. M. van de Sanden and M. N. Tsampas, *ACS Applied Materials & Interfaces*, 2019, **11**, 41267–41280.

[5] G. Zafeiropoulos, P. Varadhan, H. Johnson, L. Kamphuis, A. Pandiyan, S. Kinge, M. C. M. van de Sanden and M. N. Tsampas, *ACS Applied Energy Materials*, 2021, **4**, 9600–9610.

[6] C. X. M. Ta, C. Akamoto, Y. Furusho and F. Amano, *ACS Sustainable Chemistry & Engineering*, 2020, **8**, 9456–9463.

[7] F. Amano, H. Mukohara, H. Sato, C. Tateishi, H. Sato and T. Sugimoto, *Sustainable Energy & Fuels*, 2020, **4**, 1443–1453.

[8] K. Tsushiro, H. Sato and F. Amano, *Catalysis Today*, 2026, **461**, 115526.

[9] J. Georgieva, S. Armyanov, I. Poulios, A. D. Jannakoudakis and S. Sotiropoulos, *Electrochemical and Solid-State Letters*, 2010, **13**, P11.

[10] J. Rongé, D. Nijs, S. Kerkhofs, K. Masschaele and J. A. Martens, *Physical Chemistry Chemical Physics*, 2013, **15**, 9315–9325.

[11] F. Amano, H. Mukohara, A. Shintani and K. Tsurui, *ChemSusChem*, 2019, **12**, 1925–1930.

[12] M. Caretti, E. Mensi, R.-A. Kessler, L. Lazouni, B. Goldman, L. Carbone, S. Nussbaum, R. A. Wells, H. Johnson, E. Rideau, J.-h. Yum and K. Sivula, *Advanced Materials*, 2023, **35**, 2208740.

[13] D. O. B. Apriandanu, R. M. Surya, K. Beppu and F. Amano, *ACS Applied Energy Materials*, 2023, **6**, 10736–10741.

[14] P. P. Kunturu, S. Bera, H. Johnson and M. N. Tsampas, *Artificial Photosynthesis*, 2025, **1**, 106–116.

[15] A. Satriyatama, S. Zhou, C. Y. Toe, J. Pan, I. Facchinetti, Y. H. Ng and R. Amal, *Energy & Fuels*, 2025, **39**, 18649–18659.
